# Comparative analysis of the short-term germination and metal accumulation patterns of two *Sorghum* hybrids

**DOI:** 10.1007/s10653-025-02485-x

**Published:** 2025-04-21

**Authors:** Dávid Tőzsér, Jennifer Damilola Osazuwa, John Sule Elias, Deborah Osariemen Idehen, Daniela Isabel Gutiérrez Pérez, Ágota Zsófia Ragyák, Zsófi Sajtos, Tibor Magura

**Affiliations:** 1 Department of Ecology, University of Debrecen, Egyetem Tér 1, Debrecen, 4032 Hungary; 2 Environmental Analytical Research Group, Department of Inorganic and Analytical Chemistry, Faculty of Science and Technology, University of Debrecen, Egyetem Square 1, Debrecen, 4032 Hungary; 3 HUN-REN–UD Anthropocene Ecology Research Group, University of Debrecen, Debrecen, 4032 Hungary

**Keywords:** Sudan grass, Early-stage growth, Metal stress, Phytoremediation, Crops, Germination test

## Abstract

Metal contamination poses a high risk for organisms, especially those with extensive food chain relevancy. Thus, elevated concentration of metals is considered a major cause for concern in crops. This study aimed to evaluate the short-term responses of sorghum and Sudan grass to different Cd/Zn doses in a complex germination test by assessing growth parameters, tissue metal concentrations, and metal interaction accountant for the ecophysiological and elemental alterations. To do so, radicle and hypocotyl length were measured, and Ca, K, Mg, Cd, Cu, Fe, and Zn concentrations were determined after 24, 72, and 120 h. Our results indicated significant (*p* < 0.05) differences in the radicle and hypocotyl length by species, contaminant dose, and exposure time. Further, the applied doses along the exposure time gradient significantly and variously affected tissue concentrations. Out of the comparisons involving single metal doses, two significant interactions were revealed: the concentrations of both Cu and Fe were significantly reduced by the increase in Cd concentration in Sudan grass tissues. It was concluded that both species have an excellent potential to indicate metal contamination and accumulate metals in the short term, however, with differences in their responses along the exposure gradient. Additionally, this study filled a literature gap by revealing major patterns and limitations in growth and metal accumulation for sorghum and Sudan grass, thereby supporting further research and practical implications.

**Graphical Abstract:**

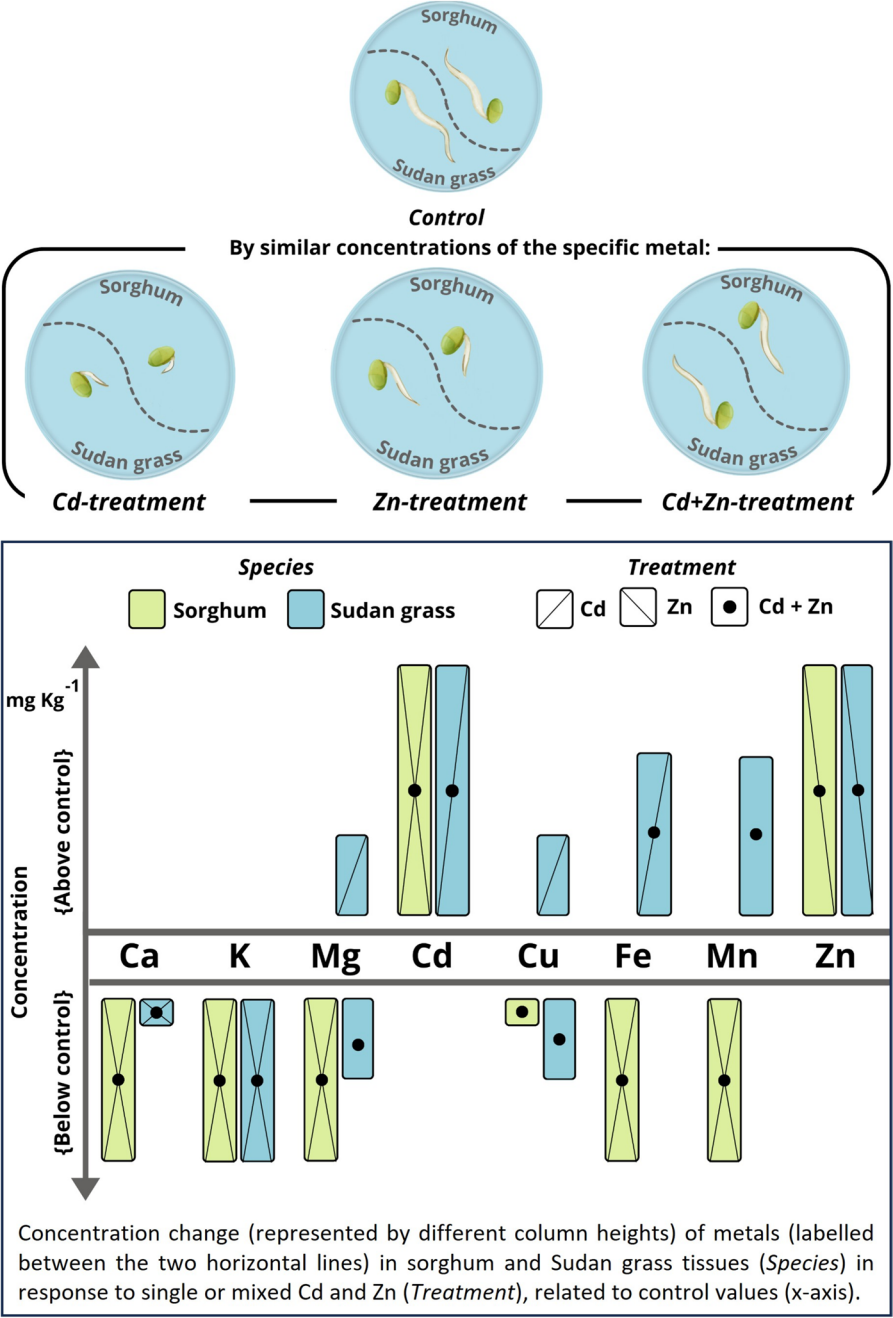

**Supplementary Information:**

The online version contains supplementary material available at 10.1007/s10653-025-02485-x.

## Introduction

The excess concentration of metals in soils affects the living conditions of individual organisms and—defined in a systematic context—also influences long-term prosperity and triggers extensive adverse mechanisms at the ecosystem level (Gautam et al., 2023). For instance, metal loads stressing plants beyond their ecological needs can widely alter their ability to reproduce successfully, which is pivotal regarding the long-term habitat occupation, which, in many cases, leads to the imbalance and disruption of food chains (Haller and Johnsson, 2020; Yadav et al., 2023). However, the agents of these phenomena cannot be considered universal threats to the environment. Based on the (semi-) natural elemental composition of soils, metals can be divided into two groups: essential and non-essential elements (González Henao & Ghneim-Herrera, 2021). According to the term itself, essential metals are basic compounds of soils due to geological, pedological, and ecological processes vital for adequate plant development (Custodio et al., 2021). This group comprises, among others, Cu, Fe, Mn, and Zn, which are all present in each soil type and required by plant species within a specific concentration range (Arif et al., 2016). On the other hand, non-essential metals barely have or have no traces under (semi-) natural conditions and are introduced from external, mostly anthropogenic sources such as agricultural substances, mistreated residues, or accidents involving metal-based compounds (Romero-Estévez et al., 2023). Non-essential metals are manifold, with Cd, Cr (VI), and Pb as major representatives from the list of the most studied ones (Alengebawy et al., 2021). This latter group is also labelled as potentially toxic elements, while their adverse ecophysiological effects can be recognized even by low soil concentrations (Sarwar et al., 2017).

It is common in both groups of metals to have a concentration threshold value above which adverse effects can be observed. It has already been well-mapped that certain metals support the emergence of various toxicity symptoms (Moulis et al., 2020). In plants, even by low (up to 1 mg kg^−1^) concentrations, Cd is known to set back the germination rate and growth of individuals along with the appearance of chlorotic leaves and the necrosis of organs (Haider et al., 2021). As for another non-essential metal, Pb also reduces germination, seedling development, and chlorophyll production based on the intensified presence of reactive oxygen species (ROS) the metal induces (Collin et al., 2022). Unlike Cd and Pb, essential metals like Mn and Zn can trigger deficiency and toxicity symptoms (Beyer et al., 2013; Zhao et al., 2012). The absence of Mn can lead to the chlorosis of leaves with necrotic spots, while excess concentrations can result in ROS-based photosynthesis damage and stunted growth (Alejandro et al., 2020; Schmidt et al., 2016). The deviation of color and size of leaves are also typical for Zn-deficiency. Its toxicity usually appears in highly reduced root and seedling growth during germination. At the same time, in mature plants, disposal and interactions with other nutrients and, thereby, extensive physiological impairments are common (Tőzsér et al., 2024; Younas et al., 2023).

The metals first reach the root as the primary organ upon contact with the polluted media. As previously evidenced by several authors, due to metabolism, the root system takes water and nutrients, whereby metals can accumulate, resulting in the uptake of non-essential elements or excess amounts of essential ones (Baker et al., 2000; Tóth et al., 2024). Regarding the fate of compounds after accumulation, metals can be transported to other plant organs where they are sequestered or, depending on the type, nature, and concentration of the pollutant, interact with different elements (Islam et al., 2024). The ratio of metal accumulation shows a highly species-dependent pattern, which observation elevates some plant species above others in this regard. The group of techniques laid on the fundamentals of this increased uptake capability is called phytoextraction (Tan et al., 2023). Previous studies have indicated that plants with the highest metal extraction potential are stress tolerant, develop an abundant deep root system, and have a high growth rate (Sharma et al., 2023; Yan et al., 2020). During the conscious application in phytoextraction, plants should fulfil additional requirements such as being a native species in the area, easy to propagate and being cost-effective and environmentally sound to maintain and harvest (Urošević et al., 2024). If all these criteria are met, phytoextraction can be considered a viable option for the remediation of metal-polluted areas. However, the lengthy period, the concentration-dependent efficiency, and the presence of polluted biomass residues from the process still support the more invasive conventional remediation technologies in many cases (Michael-Igolima et al., 2022). The elimination of these limitations is widely studied in the literature; the evolution of accumulation-promoting soil amendments, the development of biotechnological procedures, and the enhancement of follow-up biomass treatment efficiency contribute to the competitiveness of related phytotechnologies (Ferreira et al., 2022).

Based on this, several species were found to be prominent candidates in phytoextraction. Among these, *Sorghums* are thoroughly assessed in the literature in this respect. Previous studies highlighted that several members of the genus have a high degree of tolerance to stress factors like drought, heat, and metal pollution (Jawad Hassan et al., 2020; Soudek et al., 2014). These are considered great bases for survival under a broad range of environmental circumstances, but, along with these, many *Sorghum* species (e.g., *Sorghum bicolor* L.) also fulfil the criteria of being well applicable in phytoextraction; several of them show high biomass production rates and reach high tissue metal concentrations at the same time (Angelova et al., 2011; Luo et al., 2012; Sathya et al., 2016). Moreover, it was also recognized that creating hybrids from stress-tolerant species can provide new, multi-purpose alternatives like Sudan grass (*Sorghum bicolor* (L.) Moench × *Sorghum sudanense* (Piper) Stapf) having more significant potential for both agricultural and remediation use (Pupo et al., 2022; Vincze et al., 2023). This species possesses the characteristics of mother species, complemented by enhanced stress tolerance and growth rates under certain conditions (Schittenhelm and Schoetter, 2014; Li et al., 2016).

Considering the exceptional accumulation potential, the appearance of metal-toxicity symptoms is also well-known in these species. It was indicated that even low concentrations of Cd trigger responses from *Sorghum* as the antioxidant system becomes stimulated after the oxidative stress; thereby, the potential damages induced by Cd are efficiently mitigated (Liu et al., 2010). Further, in heavily Cd- and Zn-polluted soils, the species are also reported to accumulate high concentrations of the metals without showing any signs of necrosis, which is the result of the highly developed metal transportation and sequestration mechanisms between tissues (Yuan et al., 2019). The development without visible toxicity symptoms was also observed for *Sorghum bicolor* in the case of multi-contaminated soils, with parallel notable accumulation and biomass production rates (Oh et al., 2015). Therefore, the significance of *Sorghum* species in the accumulation of metals is usually raised from other perspectives; species like *S. bicolor* have substantial agricultural background in extensive areas, thus leaving no significant signs of toxicity and being harvested in contaminated soils under agricultural management can be an actual cause for concern in terms of the nutritional aspects (Blanco et al., 2016; Liaqat et al., 2024; Mbarki et al., 2022). Besides the generally extensive assessment in remediation studies, information on the early-stage evaluations of the two species is scarce (Kumar & Pathak, 2018).

This research assessed the 24-, 72- and 120 h germination and element concentration schemes of sorghum and Sudan grass in response to different single and combined Cd and Zn doses. Within this comparative analysis, it was hypothesized that (I) significant differences exist in the early radicle and hypocotyl development by dose, exposure time, and species, (II) significant changes occur in macro- and microelement concentrations also by dose, exposure time, and species, and (III) significant interactions can be observed between the accumulation of Cd/Zn and macro- and microelement after the individually supplied Cd and Zn. Testing these hypotheses was supposed to give a broad overview of sorghum and Sudan grass’s metal stress-affected development and phytoextraction potential in the early stages of their life cycle.

## Materials and methods

### Germination procedure

In the germination procedure, sorghum (*Sorghum bicolor* L.) and Sudan grass (*Sorghum sudanense* (Piper.) Stapf.) individuals (origin of seeds: Cereal Research Non-Profit Ltd., Szeged, Hungary) were involved in the same protocol. Filter papers were laid into sterile Petri dishes (⌀ 55 mm), onto which 1 ml of different solutions were dropped evenly using automated pipettes. For the test, Cd and Zn were applied as the polluting agents as 3CdSO_4_ × 8H_2_O and ZnSO_4_ × 7H_2_O (both EMSURE® ACS, purity ≥ 95%), respectively. The doses applied are presented in Table 1.

**Table 1:** Treatments used in the germination test (doses given in mg L^−1^)

Treatment type	Cd	Zn	Cd + Zn	Control
Dose (combination)	10, 50, 100, 500, 1000	10, 50, 100, 500, 1000	10 + 10, 10 + 500, 10 + 1000, 50 + 50, 50 + 100, 50 + 500, 50 + 1000, 100 + 50, 100 + 100, 100 + 500, 500 + 10, 500 + 50, 500 + 100, 500 + 500, 1000 + 10, 1000 + 50, 1000 + 1000	Distilled water

The applied doses in mixed treatments were selected to represent similar-with-similar (e.g., 100 + 100 mg L^−1^), low-with-high (e.g., 10 + 500), and high-with-low (e.g., 1000 + 50) concentrations for Cd + Zn supply schemes. The quantitative appropriateness of the applied solution was pre-assessed by wetting the filter papers with seven different (0.25, 0.5, 0.75, 1, 2, 3, and 5 mL) amounts. During this evaluation, it was found that in Petri dishes with solutions < 1 mL, the filter paper became fully dry after 96 h at the latest, causing complementary drought stress to the seedlings, while the addition of > 1 mL resulted in excess solutions remnants and the simultaneous appearance of molds by the end of the test period. Therefore, the 1 mL quantity has been selected.

After applying solutions, seven seeds were placed on the filter paper. Then, Petri dishes were capped and wrapped in aluminium foil (as the block of three dishes per package) without tilting them, thereby warranting the fixed initial distance between individual seeds. With this, the effects of light on the germination were excluded, and the competition for moisture was minimized. Dishes were put into drawers and germinated at room temperature (20.5 ± 1.4 °C). The first set of samples were germinated for 24 h, which was followed by two samplings in every 48 h (after 72 and 120 h of development). At the end of the exposure duration, the surface of the plantlets was rinsed with double distilled water to avoid the evaluation of surface metal residues. Washed individuals were laid on sterile paper sheets, and the length of the radicle and hypocotyl was measured manually with 0.1 mm of accuracy, using rulers and forcipes (type: Brand PMP) for all germinated ones. To avoid cross-contamination, rulers and forcipes were sterilized in a stem sterilizer (type: Emag Steri 15). Plantlets were then placed in lids of clean Petri dishes and kept in the oven (type: WTC Binder FD53) set at 55 °C for 48 h. Completely dried samples were homogenized in agate mortars, and the pulverized materials were put into centrifuge tubes.

### Sample digestion and determination of elemental concentrations

The sample was taken out from the tubes using plastic spatulas, and a quantity of 0.1 ± 0.01 g was measured on an analytical balance (type: Precisa ES 225SM-DR). To the recorded amount of samples, 5.0 mL HNO_3_ (65% (m/m%), reagent grade, Merck) and 1.0 mL H_2_O_2_ (30% (m/m), reagent grade, Merck) were added. The prepared solutions were then digested under high temperature (100–120 °C) and atmospheric pressure conditions. Quality control was performed using BCR-129 Hay powder reference material, with ± 10% recovery rates relative to the certified values (Anwar et al., 2025). This latter has been inserted into the revised manuscript) The digested samples were complemented to 10.00 mL of quantity with ultrapure water (Synergy UV Millipore) and kept in PP centrifuge tubes at room temperature until the next phase.

The concentration of macro- (Ca, K, Mg) and microelements (Cd, Cu, Fe, Mn, Zn) was determined by an inductively coupled plasma optical emission spectrometer (ICP-OES 5110 Vertical Dual View, Agilent Technologies) with the complementary use of a Meinhard® type nebulizer, double pass spray chamber and an autosampler (Agilent SPS4). Mono-element spectroscopic standard (1000 mg L^−1^, Scharlau) was used for the preparation of macroelement standard solution, while the microelement standards were prepared from the multi-element spectroscopic standard solution (1000 mg L^−1^, ICP IV, Merck). A 5-point calibration procedure was applied for both macro- and microelements with the dilution of standard solutions using 0.1 M HNO_3_.

### Statistical analyses

The three replications’ mean values and standard deviations were calculated to assess the changes in the radicle and hypocotyl length and elemental concentrations of sorghum and Sudan grass among the treatments and exposure intervals. Single treatments were studied and compared to the relevant results from uncontaminated (control) measurements to reveal the individual effects of Cd and Zn on growth parameters. The differences in radicle and hypocotyl length between contaminated vs. uncontaminated treatment combinations were evaluated by ANOVA and Tukey multiple comparisons of means post-hoc test.

To highlight the effects of treatments and exposure intervals on the radicle and hypocotyl length and elemental concentrations, a standardized effect size measure, the unbiased, standardized mean difference (Hedges’ *g*), was computed for the contaminated-control comparisons. This calculation was based on the following equations:

1 $$g = J\frac{{\overline{x}_{U} - \overline{x}_{C} }}{{S_{{{\text{within}}}} }}$$

2 $$S_{{{\text{within}}}} = \sqrt {\frac{{\left( {n_{U} - 1} \right)S_{U}^{2} + \left( {n_{C} - 1} \right)S_{C}^{2} }}{{n_{U} + n_{C} - 2}}}$$

3 $$J = 1 - \frac{3}{{4\left( {n_{U} + n_{C} - 2} \right) - 1}}$$

where.

$\overline{x}_{U}$ and $\overline{x}_{C}$ mean length (mm) of radicle/hypocotyl or plant element concentrations (mg kg^−1^) from uncontaminated (U) and contaminated (C) treatments;

$n_{U}$ and $n_{C}$ sample sizes for plants from uncontaminated (U) and contaminated (C) treatments;

$S_{U}$ and $S_{C}$ standard deviation for radicle/hypocotyl length or plant element concentrations from uncontaminated (U) and contaminated (C) treatments.

If the *g* values were negative, element concentration was higher in plants grown in contaminated treatments than in individuals from uncontaminated (control) ones. To study the metal accumulation and their interactions, the *esc* package was used to calculate the 95% confidence intervals (CI) of the unbiased, standardized mean difference (Hedges’ *g*) (Lüdecke, 2019). The mean effect size was found statistically significant if the 95% confidence interval did not include zero. Linear models evaluated the interaction between accumulation rates of the studied elements; thereby, synergism and antagonism could be identified between the studied elements. Statistical analyses were executed in the R program environment (version 4.1.2; R Core Team, 2018).

## Results

### Radicle and hypocotyl development of sorghum and sudan grass seedlings

**Fig. 1:**
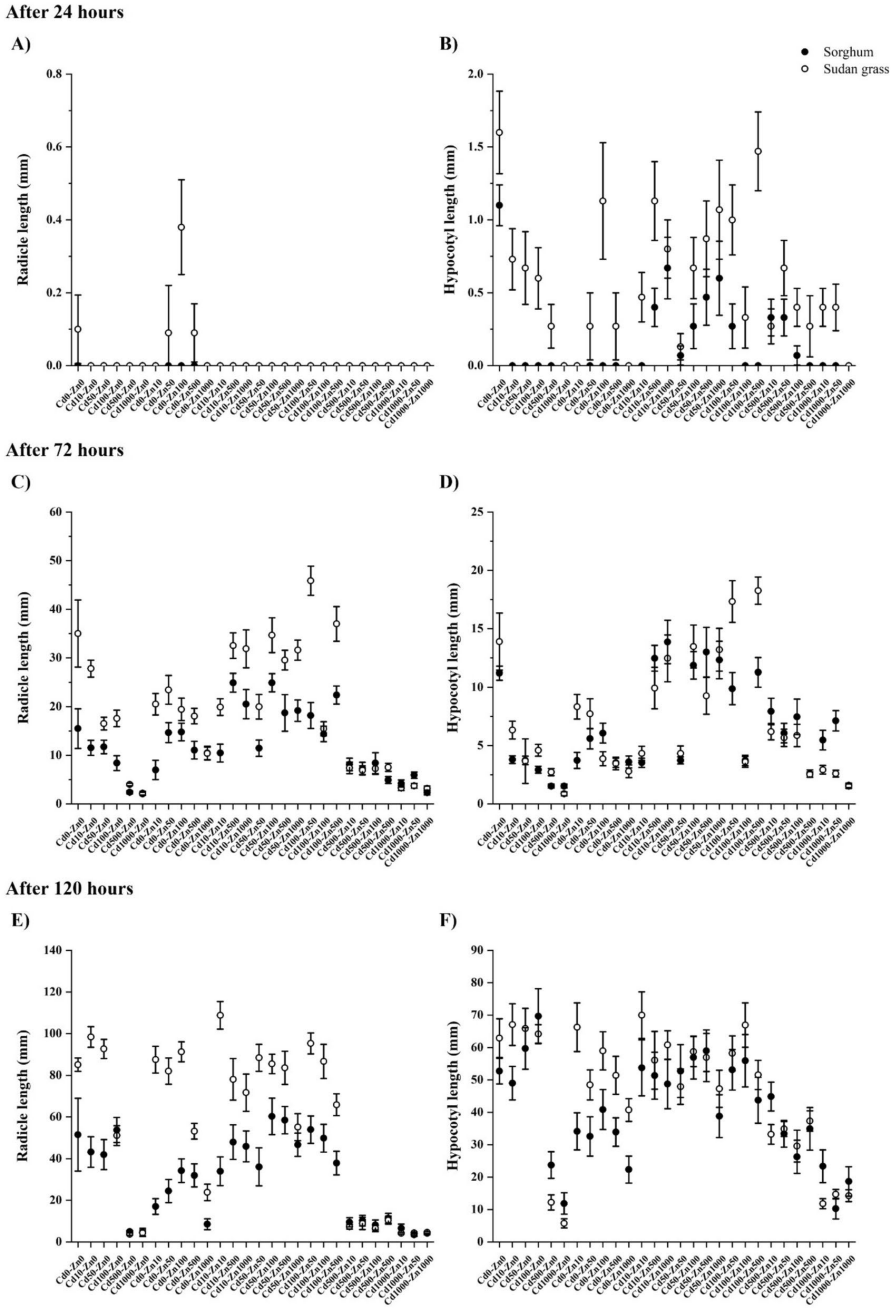
Radicle (**A, C, E**) and hypocotyl (**B, D, F**) length of sorghum (marked with ●) and Sudan grass (marked with ○) individuals after different exposure intervals (mean ± SE; *n* = 15)

Regarding growth parameters, differences in germination rate and radicle/hypocotyl lengths were found in the individual organs between the two species and the case of individual species between plant organs (Supplementary Materials A and Fig. 1). After 24 h, no radicle growth was observed besides the signs of development of only some Sudan grass seedlings in three single Zn treatments. In contrast, except for four treatments with no hypocotyl development for both species, hypocotyl germination was widespread with higher lengths for Sudan grass, with a mean value below 1.5 mm in each case.

After 72 h, radicle and hypocotyl lengths highly increased. As for the radicle, development was widely hindered by high (500 and 1000 mg L^−1^) Cd concentrations for both species, resulting in similar low lengths, while in all the other treatments, values were higher for Sudan grass than sorghum. Regarding measured hypocotyl lengths, differences between the two species were low, especially by single treatments and ones with the same Cd and Zn concentrations. Interspecific separation was also slight in treatments with various concentrations, with either species having a higher value along the treatment scheme.

After 120 h, the pattern of radicle development was very similar to that observed after 72 h, with a better general tolerance of Sudan grass than sorghum and a significant growth inhibition by high-Cd doses. Assessing the hypocotyl development, the trend demonstrated after 72 h was prevalent: differences in length were moderate, with either species being more tolerant across the individual treatments. Further, as the concentrations of Cd were increased in co-treatments, lengths heavily dropped for both species, with only negligible differences in hypocotyl development between the two.

Compared to control conditions, after 24 h, no significant effect of the treatment was found on radicle and hypocotyl growth for the species. After 72 h, the growth parameters of sorghum remained unaffected by the concentrations applied. At the same time, all the treatments containing 500 and 1 000 mg L^−1^ Cd significantly reduced Sudan grass’s radicle length, while none of the doses did influence hypocotyl length. After 120 h, sorghum radicle growth was significantly inhibited by single 10, 50, and 1 000 mg L^−1^ Zn concentrations and all the treatments containing 500 and 1 000 mg L^−1^ Cd. Sorghum hypocotyl length was reduced by 1 000 mg L^−1^ single Zn concentration and Cd500 + Zn0, Cd500 + Zn100 treatments, and the ones with 1 000 mg L^−1^ Cd. In the case of Sudan grass, radicle development was extensively influenced in many instances: related to the control values, significant inhibition was indicated by single 500 and 1 000 mg L^−1^ Zn doses, Cd10 + Zn10, Cd50 + Zn1000, Cd100 + Zn0 treatments and each one with 500 and 1 000 mg L^−1^ Cd. Also, for Sudan grass hypocotyl, a significant reduction was observed in response to added single and co-500 and 1 000 mg L^−1^ Cd, complemented by the same pattern for the single 1 000 mg L^−1^ dose (findings are based on the Tukey-test, results not presented).

### Changes in macro element concentrations in *Sorghum* and sudan grass

The different treatments only significantly influenced the concentration of tissue Ca in some instances. In the case of sorghum, Ca concentration was decreased considerably in single Zn treatments compared to control conditions in 12 out of the 15 dose-exposure time combinations. In other relations, the effects were negligible, and significant differences were sporadic (a significant increase in response to Cd500 added with Zn10 and Zn100 after 72 h and a significant decrease in response to Cd1000 added with Zn10 and Zn50 after 120 h) (Supplementary Materials B1). As for Sudan grass, in contrast, the significant influence was indicated only in one case (Cd100 + Zn50) after 72 h, while all the other doses and exposure times exerted no considerable influence on Ca patterns, shown by the means distributed on both sides of the zero value and the related tight 95% confidence intervals (Supplementary Materials B2).

The concentration of K in both species was significantly determined by the applied doses in many cases. The determining response was negative for sorghum, indicated by mean values higher than zero in most comparisons. The effects became significant after 72 and 120 h of growth in 16 control-contaminated relations. The K concentration was lower than in the control individuals in single Cd (500, 1000 mg L^−1^), single Zn (10, 50, 100, 500, 1000 mg L^−1^), and Cd + Zn (500 + 500, 1000 + 1000 mg L^−1^) treatments after 72 h and by single Cd and single Zn (both 1000 mg L^−1^) and Cd + Zn (500 + 50, 500 + 100, 1000 + 10, 1000 + 50, 1000 + 1000 mg L^−1^) doses after 120 h (Supplementary Materials B3). The degree of inhibition was also high in 13 comparisons with Sudan grass. After 72 h, tissue K concentration was significantly lower in single Cd (1000 mg L^−1^), single Zn (10, 100, 1000 mg L^−1^), and Cd + Zn (500 + 500, 1000 + 50 mg L^−1^) treatments than in control ones. After 120 h, single Cd (500, 1000 mg L^−1^), single Zn (10 mg L^−1^), and Cd + Zn (50 + 100, 500 + 500, 1000 + 50, 1000 + 1000 mg L^−1^) were adequate to reduce the concentration of K significantly (Supplementary Materials B4).

In the case of Mg, contrary trends were observed between sorghum and Sudan grass. For sorghum, 27 significant comparisons were found, each with the negative influence of the added dose on the concentration of Mg. Single Cd (10, 50, 500, 1000 mg L^−1^), single Zn (10, 50, 100, 500, 1000 mg L^−1^), and Cd + Zn (10 + 10, 10 + 500, 10 + 1000, 50 + 500, 50 + 1000, 100 + 50, 100 + 500, 100 + 100, 500 + 500, 1000 + 10, 1000 + 1000) treatments were effective after 72 h, while after 120 h, some single Zn (10, 1000 mg L^−1^) and Cd + Zn (500 + 50, 500 + 100, 1000 + 10, 1000 + 50, 1000 + 1000 mg L^−1^) were also identified as significant agents in decreasing tissue Mg in sorghum (Supplementary Materials B5). On the other hand, in Sudan grass, two significant positive (single 10 and 50 mg L^−1^ Cd after 120 h) and four significant negative (Cd1000 added with Zn0, Zn 10, Zn50, and Zn1000 after 120 h) responses were demonstrated after the addition of metal doses and besides these previous, mean values compared to the control concentrations deviated in both directions (Supplementary Materials B6).

### Changes in microelement concentrations in *Sorghum* and sudan grass

The applied doses significantly influenced the Cd concentration of both species. In the case of sorghum, only the single Zn had no significant effect on the Cd concentrations; all the other treatments increased the tissue Cd significantly. The highest differences compared to control individuals were observed in Cd1000 + Zn50 after 120 h, Cd500 + Zn100 and Cd100 + Zn50 after 24 h, and Cd50 + Zn500 and Cd50 + Zn1000 after 72 h, even higher than in treatments of single Cd doses. In the case of Sudan grass, similar to sorghum, single Zn doses did not alter the concentration of Cd in individuals. Further, all the other treatments elevated tissue Cd significantly. The highest accumulation intensity was in treatments Cd50 + Zn50 and Cd1000 + Zn0 after 120 h of development, while Cd50 + Zn100, Cd50 + Zn0, and Cd1000 + Zn0 were also doses with very high accumulation rates, indicating no distinct differentiation in the Cd accumulation performance of Sudan grass between single and Cd + Zn treatments.

In the case of Cu, significant effects of the doses and exposure times on the species’ metal concentration were detected only in very few comparisons. In contrast, the mean effect sizes were distributed on both sides of the zero-value line within a relatively confined Hedges’ *g* value range. Regarding sorghum, a significant negative influence was found only for the Cd100 + Zn50 treatment after 72 h of growth, indicating the mitigating effect of the specific co-treatment on the tissue Cu concentration. For Sudan grass individuals, three significant relations were revealed. Single 100 mg L^−1^ Cd significantly increased the Cu concentration in studied plantlets after 72 h and single 10 mg L^−1^ Cd after 120 h. Contrarily, the simultaneous supply of 1000 mg L^−1^ Cd and Zn significantly decreased the concentration of Cu after 120 h.

The prevailing trends in the Fe concentration were opposite between sorghum and Sudan grass, with several significant comparisons. In the case of sorghum, 89% of the comparisons were in the positive range, meaning, at the same time, that, compared to control individuals, the applied doses decreased the tissue Fe concentration in all the significant relations (*n* = 27). After 72 h, the influence was significant for the single 50, 500, and 1000 mg L^−1^ Cd, all the single Zn, and Cd10 + Zn10, Cd10 + Zn500, Cd10 + Zn1000, Cd50 + Zn500, Cd50 + Zn1000, Cd100 + Zn50, Cd100 + Zn100, Cd500 + Zn500, Cd1000 + Zn10 treatments. After 120 h, a significant influence of single 1000 mg L^−1^ Cd and single 10 and 1000 mg L^−1^ Zn was found, while Cd10 + Zn10, Cd50 + Zn50, Cd500 + Zn10, Cd500 + Zn50, Cd1000 + Zn10, Cd1000 + Zn50, and Cd1000 + Zn1000 were also influential in significantly decreasing Fe in sorghum. For Sudan grass, on the other hand, 68% of the comparisons were found in the negative range, and eight out of the nine significant comparisons indicated an increase in Fe concentration after the addition of metals. In detail, the Fe concentration of plantlets was significantly higher than that of the control ones in the Cd10 + Zn10, Cd10 + Zn1000, and Cd50 + Zn500 treatments after 72 h, and in the Cd10 + Zn0, Cd50 + Zn0, Cd10 + Zn1000, Cd50 + Zn50, and Cd100 + Zn100 treatments after 120 h. In contrast, the Cd1000 + Zn50 treatment significantly decreased Fe in tissues after 120 h, compared to control individuals.

The concentration patterns for Mn also showed differences between the two species. In the case of sorghum, 84% of the Hedges’ *g* values and all the significant relations (*n* = 24) were in the positive range, reflecting the general negative (concentration-decreasing) effect of the applied doses on Mn flux. Single 100 mg L^−1^ Zn after 24 h, single 10, 50, 500, and 1000 mg L^−1^ Cd, all the single Zn, and Cd10 + Zn10, Cd10 + Zn1000, Cd100 + Zn50, Cd100 + Zn100, Cd100 + Zn500, Cd500 + Zn500, Cd1000 + Zn1000 treatments after 72 h were all significantly influential, while single 10 and 1000 mg L^−1^ Zn, Cd500 + Zn10, Cd500 + Zn50, Cd500 + Zn100, Cd1000 + Zn10, and Cd1000 + Zn50 significantly decreased tissue Mn after 120 h. As for Sudan grass, the distribution of mean effect size values was even between the negative and positive ranges, while all the six significant relations (Cd0 + Zn1000, Cd500 + Zn50. Cd500 + Zn500, Cd1000 + Zn10, Cd1000 + Zn50, Cd1000 + Zn1000, each after 120 h) were ones decreasing Mn in plantlets.

The changes in Zn concentration across the treatments were significant for both species in most cases. The effects of single Cd doses were insignificant for sorghum except for one comparison (500 mg L^−1^ decreasing tissue Zn after 72 h). Additionally, except for the 10 mg L^−1^ treatment, single Zn doses significantly increased Zn in plantlets, with the highest difference to the control plants (among all treatments) in the 500 mg L^−1^ treatment after 120 h. Among the 51 Cd + Zn dose-exposure time combinations, 37 significant cases were found. The highest differences to control plants (the most negative Hedges’ *g* values) were indicated for Cd50 + Zn500, Cd50 + Zn1000, Cd500 + Zn100, and Cd1000 + Zn1000 after 72 h. Insignificant relations for Cd + Zn treatments were observed primarily by low (10 and 50 mg L^−1^) Zn supplies. For Sudan grass, the effects of single Cd doses were also insignificant, except for one comparison (100 mg L^−1^ increasing tissue Zn after 72 h). Apart from the negligible effect of Cd0 + Zn10 after 24 h, all the single Zn treatments resulted in a significant Zn concentration increase in tissues, with the highest values for 1000 mg L^−1^ after 24 and 72 h. Except for three doses, all the combined treatments elevated seedling Zn concentration significantly. The highest values (among all treatments) were found for Cd50 + Zn1000 after 120 h and Cd500 + Zn500 after 72 h.

### Interaction between the concentrations of metals

After assessing the responses of plant element concentrations to different doses and exposure times, the interactions between the single metal (Cd and Zn) supply-based concentrations of Cd and Zn and the target elements in tissues were also investigated for sorghum and Sudan grass. Two significant correlations were identified after running the 28 pairwise comparisons (Supplementary Materials C1 and C2).

**Fig. 2:**
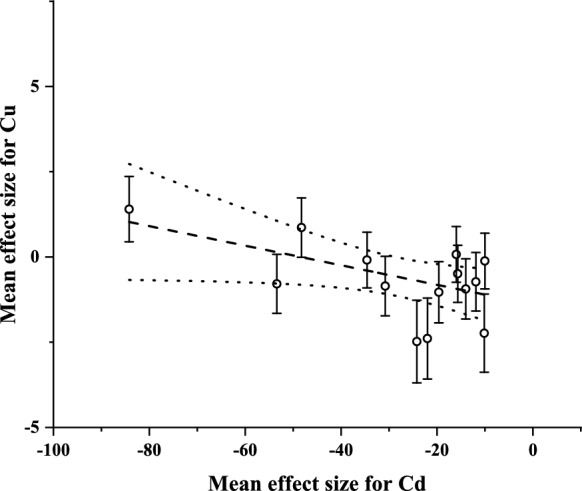
Interaction between Cd and Cu accumulation in Sudan grass based on the relationship between the standardized mean difference (Hedges’ *g*) values (mean ± SE of the effect size for Cu) from single Cd-contaminated vs. uncontaminated comparisons. The calculations were performed with the pooled mean values of 24-, 72- and 120 h treatments (weighted linear regression: *F* = 4.7471, *n* = 15, *p* = 0.0483, *R* = − 0.5172). Dotted line: 95% confidence interval

In the first case, a significant negative correlation was found in the mean effect size values for Cd and Cu in Sudan grass. It means that increased Cd concentration in tissues significantly decreased the concentration of Cu in plantlets (Fig. 2.)

**Fig. 3:**
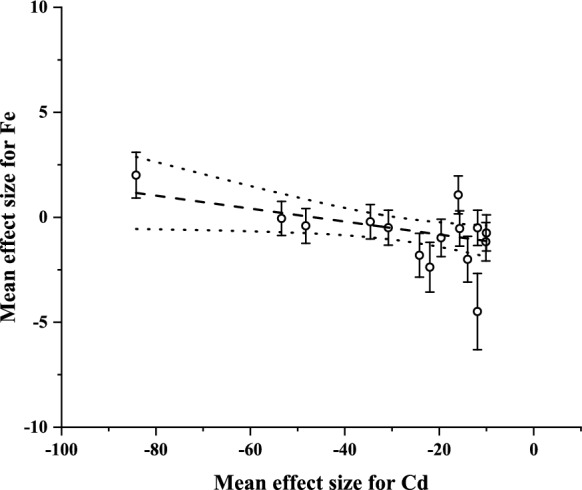
Interaction between Cd and Fe accumulation in Sudan grass based on the relationship between the standardized mean difference (Hedges’ *g*) values (mean ± SE of the effect size for Fe) from single Cd-contaminated vs. uncontaminated comparisons. The calculations were performed with the pooled mean values of 24-, 72- and 120 h treatments (weighted linear regression: *F* = 5.3879, *n* = 15, *p* = 0.0372, *R* = − 0.5413). Dotted line: 95% confidence interval

The other significant relationship was also a significant negative correlation between the mean effect size values for Cd and Fe in Sudan grass. This correlation indicates that increasing tissue Cd concentration decreased the concentration of Fe in the studied plantlets (Fig. 3.)

## Discussion

The results proved our first hypothesis as major differences were found regarding the radicle and hypocotyl growth by species, contaminant dose, and exposure time. In the literature, there is no comprehensive evidence on such aspects of the short-term germination performance of *Sorghum* species presented in this research. Further, despite the extensive approach to address the element accumulation patterns of *Sorghum* species in response to metal stress, the in-depth early-stage revision of radicle and hypocotyl growth along Cd and Zn pollution- and exposure time gradients are scarce. By assessing the Cd-influenced sorghum development (*Sorghum bicolor*), Zhan et al. (2017) observed a significant reduction in radicle growth in response to increasing Cd supply. The authors attributed this to the severely altered homeostasis of ROS and auxin. Further, Liu et al. (2010) underlined that high (> 50 mg kg^−1^) soil Cd concentrations widely decreased the activity of four substantial antioxidants in both sorghum and Sudan grass, which, upon contact, could have general adverse effects on the development in each growth stage, which also support the findings shown here. On the other hand, Ertekin et al. (2020) performed a 10-day-long experiment. Unlike those presented after 24 h in this paper, the authors found no major differences in germination rates among the different (0–800 mg L^−1^) doses applied. However, after 72 and 120 h, this study demonstrated high (≥ 80%) germination rates for both species in the single and combined treatments, which is in accordance with the results from Pandian et al. (2020), who found highly evolved adaptation mechanisms against metal loads in sorghum, especially in cases of their combined presence. This latter can explain the outcomes presented here for germination lengths; in co-treatments with high Cd concentration, growth performance was generally better than with high single Cd concentrations, suggesting the alleviating effect of Zn on Cd toxicity. In general, Rizwan et al. (2019) explained this afore observation of our study by the role of Zn in counteracting the Cd-based ROS formation by elevating the activity of antioxidant enzymes. The role of Zn in young sorghum plants was also revealed by Maharajan et al. (2023). The authors indicated that root and shoot lengths were highly reduced in the case of Zn deficiency, which confirmed the recognition of a threshold value below which the metal is considered a limiting factor. It was also proved in our study by finding higher radicle and hypocotyl lengths in response to elevating the concentration of Zn in single treatments in most of the comparisons. On the other hand, the adverse effects of Zn have also been reported in the literature. Usha Shri et al. (2017) evidenced a gradual decrease in root and shoot length and chlorophyll content in sorghum seedlings treated with elevated (0–7.5 mM) single Zn concentrations after ten days of exposure. The authors attribute this observation to the phytotoxic effect of Zn manifested by insufficient chlorophyll-b. Regarding the contrary findings on the role of Zn in plant development, Hassan et al. (2022) concluded that the toxic nature of the metal is dependent on several factors, such as species, time of exposure, or test setup features, having the potential to trigger adverse ecophysiological consequences even by low concentrations. This is supported by the interspecific differences in our study for sorghum and Sudan grass, especially in moderate doses. These inherent differences in growth can be further enhanced by genetic variation; Badigannavar et al. (2016) highlighted that specific breeding practices can lead to an alteration in elemental composition and growth of individual *Sorghum* species, providing a further variable accountant for the differences between the results of individual studies with similar designs.

Our second hypothesis was also supported as the metal concentrations were significantly altered in the species by the applied dose, with differences also by exposure time. In close association with the second hypothesis, the third hypothesis was based on the interactions between elements assessed by linear regression. This hypothesis was fulfilled only in a few instances since significant interaction between the concentration of either Cd or Zn and that of other tissue elements was found only in two out of the 28 comparisons. Regarding the studied macro elements, it was found that single Zn doses significantly decreased. At the same time, co-treatments variously affected the tissue concentration of Ca in sorghum, while that of Sudan grass was only slightly influenced by the metals. According to Prasad et al. (2016), an antagonism exists between Ca and Zn by the root accumulation, which results in contrary concentration trends for the two elements in most cases. The interactions between Cd/Zn doses and macro elements in *Sorghum* species are rarely addressed in the literature. However, as indicated above, depending on the concentrations, Zn can have a vital role in buffering the adverse effects of Cd in crops directly by supporting the antioxidative system or indirectly by the antagonistic accumulation based on their similar ionic radii, whereby the adequate macro nutritional (e.g., Ca) status of tissues can also be protected (Zhou et al., 2020). As for the difference found between species in this study, Zheng et al. (2024) indicated that *Sorghum* species have a very high degree of interspecific and genotype-based difference regarding the macro- and microelemental composition patterns, which are in association with general stress tolerance and metal interaction schemes.

Unlike for Ca, the concentration of K was extensively and significantly decreased for both species, primarily by single and co-treatments with high concentrations. In contrast with the findings here, Benáková et al. (2017) reported no antagonism between single and co-Cd/Zn doses and tissue K concentration in *Brassica napus*. Besides this previous observation, the prevailing relations in the literature differ. Widespread antagonism was reported between K and Cd by Wang et al. (2023); however, in a reverse approach, referring to K as an element being able to reduce both the concentration and the toxic effects exerted by Cd. Siddiqui et al. (2012) also emphasized that metal loads induced a decrease in tissue K. Considering Zn, the moderate concentrations were found to favor K balance (Ali Raza et al., 2021; Chaudhary et al., 2017), which is not supported in this research; Zn addition was not able to support tissue K in all single treatments, while it was also not efficient to offset the adverse effects of Cd in combined ones. This can be explained by the fact that *Sorghum* species and genotypes are considered sensitive to abiotic stress factors in the early stages of development. Thus, external metal supply can lead to severe nutritional imbalance (Djanaguiraman et al., 2014; Mbarki et al., 2022).

Similar trends were observed in the case of sorghum Mg, with generally significant concentration drops in response to Cd/Zn doses. Regarding Sudan grass, interactions were various. In the literature, findings are also quite contrasting and highly vary by species. Hermans et al. (2011) reported that low *Arabidopsis* tissue Mg concentrations indirectly compensated for the toxic effects of Cd, while for Chinese cabbage, Lu et al. (2021) highlighted the role of Mg addition in plants by alleviating Cd-related health issues; this latter was also concluded by Nazar et al. (2012) by naming the better antioxidant status supported by excess Mg as the main reason. From the reverse perspective, Wyszkowski (2002) and Ciećko et al. (2005) found that Cd in the growing media enhanced the concentration of Mg in plant tissues, corresponding to the results found here for Sudan grass after 120 h. Added, supporting the findings here for sorghum, Fu et al. (2024) demonstrated that Zn significantly hindered the accumulation of Mg in rice plants due primarily to the competition for the same transport pathways. It might explain the significant negative correlations found in treatments including Zn for both species.

Regarding the studied microelements, it was found that added Cd had a significantly positive correlation with tissue Cd concentration. For sorghum, the highest accumulation rates were found for combined treatments, while such a difference did not exist for Sudan grass. Kuriakose and Prasad (2019) presented that the interaction between Cd and Zn in sorghum is highly variable and dependent on the concentration of metals. It is evident from our results that high tissue Cd concentrations were reached by low Cd paired with high Zn and high Cd paired with low Zn doses. Studying *Festuca arundinacea*, Dong et al. (2019) also observed mixed effects of Zn on tissue Cd concentrations, depending on the plant organ and the level of contamination. Various effects and also the less sensitive character of Sudan grass to the presence of Zn by Cd accumulation may be attributed to the fact that, despite being an essential element, there is significant variability in responses to the presence of Zn among different species, which can also widely influence the elemental (e.g., Cd) patterns as well (Noulas et al., 2018).

Unlike for Cd, the concentration of Cu was less dependent on the Cd and Zn doses, with only a few significant comparisons. In the available literature, there is no in-depth evidence on the effects of Cd and Zn on Cu concentration in *Sorghum*. However, some information is available on other species. For instance, Haider et al. (2021) indicated that Cd highly reduces the concentration of microelements like Cu in plants, which corresponds to the observations found in this study for sorghum and Sudan grass in the latter species also confirmed by a linear regression-based significant negative correlation in single Cd treatments between tissue Cd and Cu of individuals. Drawing the same conclusion, Gussarsson et al. (1995) proposed that ions with similar characteristics (e.g., valency) may readily compete by their accumulation, which can be accounted for an antagonistic relationship in tissues. Additionally, Behtash et al. (2022) revealed that Zn significantly improved the condition of *Cucurbita pepo* under Cu stress. In contrast, Luo and Rimmer (1995) found that the increased Zn toxicity was aggravated by adding Cu, assuming a synergism between the two elements in this regard. For Sudan grass, opposing trends were presented only by the very high-concentration combined treatment, with presumed synergism-based disruptive effects on enzymatic processes related to Cu (Soni et al., 2024).

In the case of Fe, a contrary pattern was found between the two species; most of the applied doses decreased the Fe concentration in sorghum individuals, while in Sudan grass, concentration increased in the majority of instances, with significant differences in many comparisons. Contrary to the previous results for Sudan grass, a significant negative correlation was indicated between tissue Cd and Fe concentrations in single Cd treatments according to the linear regression. It suggests that the Cd enrichment of tissues was not concurrent with the increased concentration in the added dose (as presented in Supplementary materials B8), which resulted in very high tissue Cd concentrations even by relatively lower doses, thereby impairing the Fe balance. Similarly, Zhang et al. (2023) reported a significant decrease in the Fe concentration of rice individuals in response to the Cd supply, leading to a considerable setback in plants’ condition. Similar antagonism was indicated by Biyani et al. (2019), who underlined that the antioxidant enzyme activity was also highly impeded in the absence of Fe. The effects of Zn on Fe concentration have been scarcely studied so far; however, a negative general interaction/inhibition is supposed between the two in the literature (Hanikenne & Bouché, 2023). These findings above are in association with the ones introduced for sorghum. The increased Fe concentration in Sudan grass compared to control individuals is an unprecedented phenomenon, which, at the same time, provides the species a remarkable position in early-growth experiments by underlining the ability to maintain a good Fe balance even under severe metal stress.

The trends in Mn were very similar between the two species, with the general indication of decreasing concentrations after adding Cd/Zn doses, which were complemented by some significant relations. Following these, Mawouma et al. (2022) proposed a competitive antagonism between Zn and Mn in the evaluated sorghum individuals. Moreover, Adamczyk-Szabela et al. (2020) noted that both Cd and Zn reduced the concentration of Mn in *Melissa officinalis* roots, suggesting a competition between them for the same transporters. Further, Zn is also known as a metal that can disturb the homeostasis of several elements, among other Mn, lowering its concentration in the studied plants (Zhao et al., 2012). Knowing that it is considered an essential and frequently reported mechanism by organisms (Eijkelkamp et al., 2014), the lower concentrations in this paper compared to control sorghum and Sudan grass individuals can be traced back to this previous and well-mapped pattern.

For tissue Zn, most of the Zn treatments increased its concentration significantly in sorghum and Sudan grass. The highest concentration differences to control individuals were revealed in combined treatments after 72 h for both species. Apart from extremely high concentrations in the media, the effects of external Zn supply on tissue Zn concentration are explored and confirmed by positive correlations in most cases. Still, information on the influence of single Cd and combined Cd + Zn doses on internal Zn concentration remains inconsistent (Kuriakose & Prasad, 2019). In many studies, antagonism has been reported; Tkalec et al. (2014) observed that Cd decreased the Zn accumulation intensity in tobacco. Contrarily, Cherif et al. (2011) concluded that high Cd and Zn concentrations in tomatoes have a synergistic effect, which amplifies the process intensities (e.g., stress symptoms and accumulation mechanisms) compared to the presence of only one of them. Furthermore, Almeida et al. (2019) demonstrated an increased Zn accumulation in treatments with elevated combined Cd + Zn concentrations than in ones with lower doses. These references on the role of combined effects may support the fact that the most intensive Zn accumulation by the two species was found in Zn treatments, including Cd, in this study.

## Conclusions

This study evaluated the early-stage growth and metal concentration schemes in sorghum and Sudan grass plantlets in response to different Cd and Zn doses. Major practical implication characteristics have been introduced as both the contaminant dose and exposure time influenced the growth of radicle and hypocotyl of the species significantly; high concentration Cd treatments were more limiting than highly elevated Zn doses, while a moderating effect of Zn was indicated when applied together with Cd. In most cases, Sudan grass had higher radicle and hypocotyl lengths than sorghum.

In the case of tissue element concentrations, significant applied treatment- and concentration-dependency were also indicated. Among macro elements, K concentration was significantly decreased in both species compared to control results. In contrast, the drop in concentration was significant for Ca and Mg only in sorghum, primarily after 72 and 120 h. Among microelements, Cd and Zn concentrations were strongly associated with the applied doses, with species showing the highest co-treatment accumulation rates. As for Fe and Mn, contrary trends were found for the two species; most of the treatments resulted in a significant decrease in the concentration of both metals in sorghum, while, in more than two-thirds of the treatments, Fe and Mn concentration increased significantly in Sudan grass, with 72- and 120 h plantlets being the most affected ones. The applied treatments barely influenced the concentration of Cu.

From the 28 single-treatment comparisons, only two significant tissue element correlations were identified. The concentrations of Cu and Fe were significantly decreased in response to the increase of Cd in Sudan grass individuals. As this relation was revealed by single-Cd treatments, the sole application involving all such concentrations by exposure-time pooled values, sole Cd contamination is regarded as a major concern with extensive effect mechanism in Sudan grass.

These findings indicate that different dose-exposure time combinations significantly affect the growth and element concentrations of sorghum and Sudan grass. The experiment was run under controlled conditions; however, general early-stage responses should also be considered in field tests and cultivation practices. Since both species have considerable agricultural relevance, seeding in Cd- and Zn-contaminated areas can cause concern; germination deficiencies and visible toxicity symptoms on plantlets can emerge under certain conditions, while the potential pursuant production of contaminated biomass suggests a food chain-related threat. At the same time, further research is needed with various test setups to determine plant characteristics and related accumulation-based features more extensively.

## Supplementary Information

Below is the link to the electronic supplementary material.

Supplementary file1 (DOCX 869 KB)

## Data Availability

No datasets were generated or analysed during the current study.
