# Supplementary material for: Comparative analysis of the short-term germination and metal accumulation patterns of two Sorghum hybrids
Source: Environ Geochem Health. 2025 Apr 21;47(5):178. doi: 10.1007/s10653-025-02485-x (PMC12011658; doi:10.1007/s10653-025-02485-x)
Supplement: Supplementary file 1 — Supplementary file1 (DOCX 869 KB) [file 10653_2025_2485_MOESM1_ESM.docx]

**Supplementary Materials A**. Germination performance of sorghum and Sudan grass seedlings in different treatments after 24-, 72- and 120 hours of development.

| **Treatment** | **Germination rate (%)** | | | | | |
| --- | --- | --- | --- | --- | --- | --- |
|  | **After 24 hours** | | **After 72 hours** | | **After 120 hours** | |
|  | Sorghum | Sudan grass | Sorghum | Sudan grass | Sorghum | Sudan grass |
| ***Single Cd*** |  |  |  |  |  |  |
| Cd10 | 0.00 ± 0.00 | 33.33 ± 6.67 | 100.00 ± 0.00 | 10.00 ± 0.00 | 100.00 ± 0.00 | 100.00 ± 0.00 |
| Cd50 | 0.00 ± 0.00 | 26.67 ± 6.67 | 100.00 ± 0.00 | 100.00 ± 0.00 | 86.67 ± 0.00 | 100.00 ± 0.00 |
| Cd100 | 0.00 ± 0.00 | 26.67 ± 6.67 | 93.33 ± 6.67 | 100.00 ± 0.00 | 93.33 ± 6.67 | 100.00 ± 0.00 |
| Cd500 | 0.00 ± 0.00 | 13.33 ± 6.67 | 86.67 ± 6.67 | 100.00 ± 0.00 | 93.33 ± 6.67 | 86.67 ± 6.67 |
| Cd1000 | 0.00 ± 0.00 | 6.67 ± 6.67 | 100.00 ± 0.00 | 93.33 ± 6.67 | 100.00 ± 0.00 | 100.00 ± 0.00 |
| ***Single Zn*** |  |  |  |  |  |  |
| Zn10 | 0.00 ± 0.00 | 0.00 ± 0.00 | 100.00 ± 0.00 | 86.67 ± 6.67 | 100.00 ± 0.00 | 100.00 ± 0.00 |
| Zn50 | 0.00 ± 0.00 | 6.67 ± 6.67 | 100.00 ± 0.00 | 86.67 ± 6.67 | 93.33 ± 6.67 | 100.00 ± 0.00 |
| Zn100 | 0.00 ± 0.00 | 6.67 ± 6.67 | 86.67 ± 6.67 | 100.00 ± 0.00 | 100.00 ± 0.00 | 100.00 ± 0.00 |
| Zn500 | 0.00 ± 0.00 | 6.67 ± 6.67 | 100.00 ± 0.00 | 100.00 ± 0.00 | 93.33 ± 6.67 | 100.00 ± 0.00 |
| Zn1000 | 0.00 ± 0.00 | 0.00 ± 0.00 | 86.67 ± 6.67 | 100.00 ± 0.00 | 66.67 ± 17.64 | 100.00 ± 0.00 |
| ***Cd+Zn*** |  |  |  |  |  |  |
| Cd10+Zn10 | 0.00 ± 0.00 | 26.67 ± 6.67 | 86.67 ± 6.67 | 100.00 ± 0.00 | 100.00 ± 0.00 | 100.00 ± 0.00 |
| Cd10+Zn500 | 40.00 ± 11.55 | 53.33 ± 17.64 | 100.00 ± 0.00 | 93.33 ± 6.67 | 100.00 ± 0.00 | 100.00 ± 0.00 |
| Cd10+Zn1000 | 46.67 ± 13.33 | 40.00 ± 20.00 | 93.33 ± 6.67 | 93.33 ± 6.67 | 100.00 ± 0.00 | 100.00 ± 0.00 |
| Cd50+Zn50 | 6.67 ± 6.67 | 13.33 ± 6.67 | 100.00 ± 0.00 | 100.00 ± 0.00 | 93.33 ± 6.67 | 100.00 ± 0.00 |
| Cd50+Zn100 | 10.00 ± 11.55 | 26.67 ± 6.67 | 100.00 ± 0.00 | 100.00 ± 0.00 | 100.00 ± 0.00 | 100.00 ± 0.00 |
| Cd50+Zn500 | 33.33 ± 6.67 | 26.67 ± 6.67 | 80.00 ± 11.55 | 100.00 ± 0.00 | 93.33 ± 6.67 | 100.00 ± 0.00 |
| Cd50+Zn1000 | 33.33 ± 13.33 | 33.33 ± 6.67 | 100.00 ± 0.00 | 100.00 ± 0.00 | 86.67 ± 6.67 | 100.00 ± 0.00 |
| Cd100+Zn50 | 20.00 ± 11.55 | 33.33 ± 6.67 | 100.00 ± 0.00 | 100.00 ± 0.00 | 100.00 ± 0.00 | 100.00 ± 0.00 |
| Cd100+Zn100 | 0.00 ± 0.00 | 13.33 ± 6.67 | 100.00 ± 0.00 | 100.00 ± 0.00 | 93.33 ± 6.67 | 100.00 ± 0.00 |
| Cd100+Zn500 | 0.00 ± 0.00 | 46.67 ± 17.64 | 100.00 ± 0.00 | 100.00 ± 0.00 | 93.33 ± 6.67 | 100.00 ± 0.00 |
| Cd500+Zn10 | 33.33 ± 13.33 | 13.33 ± 6.67 | 93.33 ± 6.67 | 100.00 ± 0.00 | 100.00 ± 0.00 | 100.00 ± 0.00 |
| Cd500+Zn50 | 33.33 ± 6.67 | 40.00 ± 20.00 | 100.00 ± 0.00 | 100.00 ± 0.00 | 93.33 ± 6.67 | 100.00 ± 0.00 |
| Cd500+Zn100 | 6.67 ± 6.67 | 26.67 ± 6.67 | 93.33 ± 6.67 | 100.00 ± 0.00 | 93.33 ± 6.67 | 100.00 ± 0.00 |
| Cd500+Zn500 | 0.00 ± 0.00 | 6.67 ± 6.67 | 100.00 ± 0.00 | 100.00 ± 0.00 | 100.00 ± 0.00 | 100.00 ± 0.00 |
| Cd1000+Zn10 | 0.00 ± 0.00 | 26.67 ± 6.67 | 93.33 ± 6.67 | 100.00 ± 0.00 | 80.00 ± 11.55 | 100.00 ± 0.00 |
| Cd1000+Zn50 | 0.00 ± 0.00 | 20.00 ± 11.55 | 100.00 ± 0.00 | 93.33 ± 6.67 | 80.00 ± 0.00 | 100.00 ± 0.00 |
| Cd1000+Zn1000 | 0.00 ± 0.00 | 0.00 ± 0.00 | 100.00 ± 0.00 | 100.00 ± 0.00 | 93.33 ± 6.67 | 100.00 ± 0.00 |
| **Control** | 46.67 ± 17.64 | 93.33 ± 6.67 | 86.67 ± 6.67 | 100.00 ± 0.00 | 100.00 ± 0.00 | 100.00 ± 0.00 |

**Supplementary materials B1**. Mean effect sizes (mean Hedges’ *g* ± 95% CI) for the accumulation of Ca in sorghum plantlets in different treatments (mg kg^-1^) after different exposure times (hours). Negative *g* values indicate higher concentration in individuals grown in contaminated vs. ones from uncontaminated media. The mean effect size was considered statistically significant if the 95% bootstrap confidence interval (CI) did not include zero.


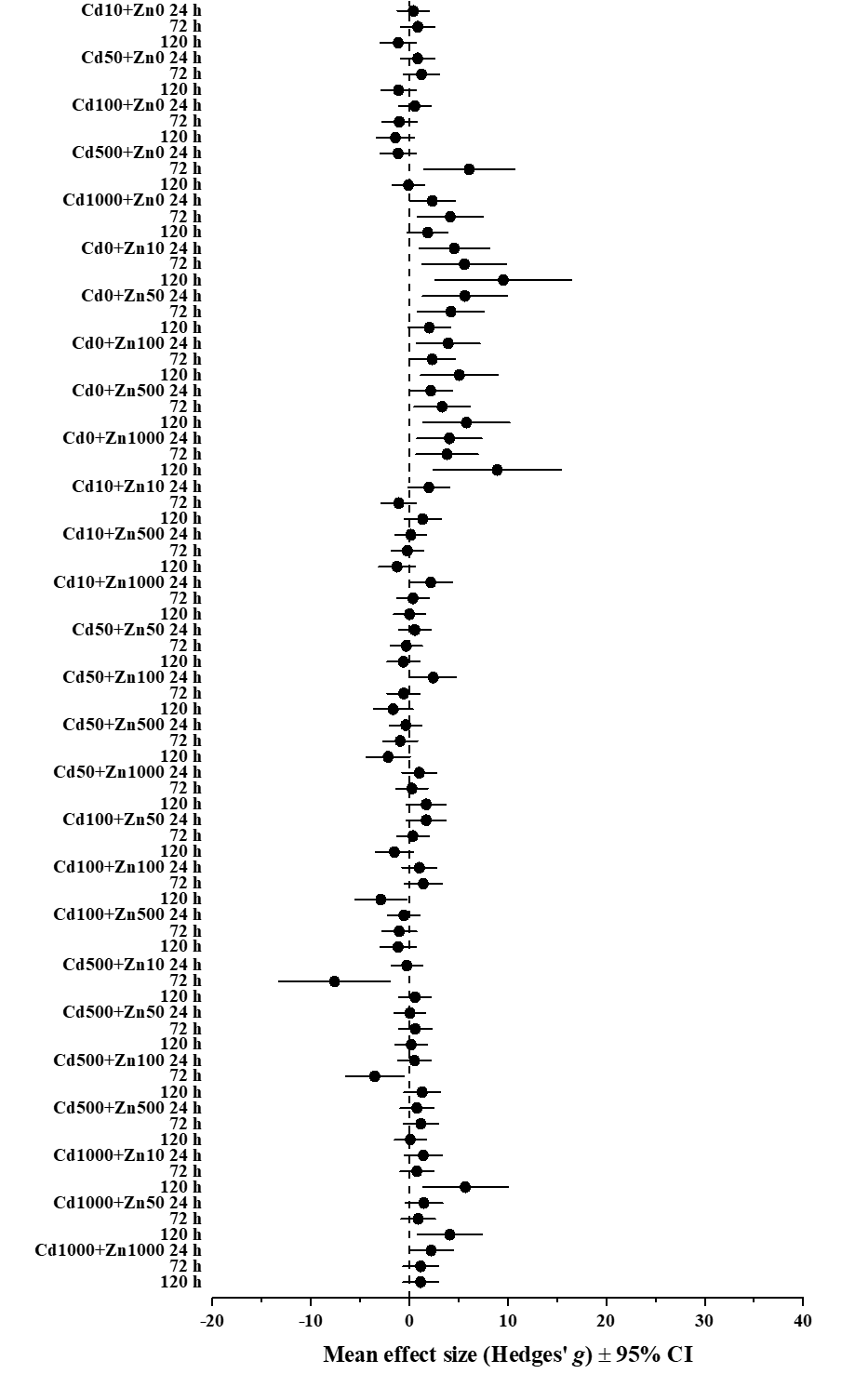


**Supplementary materials B2**. Mean effect sizes (mean Hedges’ *g* ± 95% CI) for the accumulation of Ca in Sudan grass plantlets in different treatments (mg kg^-1^) after different exposure times (hours). Negative *g* values indicate higher concentration in individuals grown in contaminated vs. ones from uncontaminated media. The mean effect size was considered statistically significant if the 95% bootstrap confidence interval (CI) did not include zero.


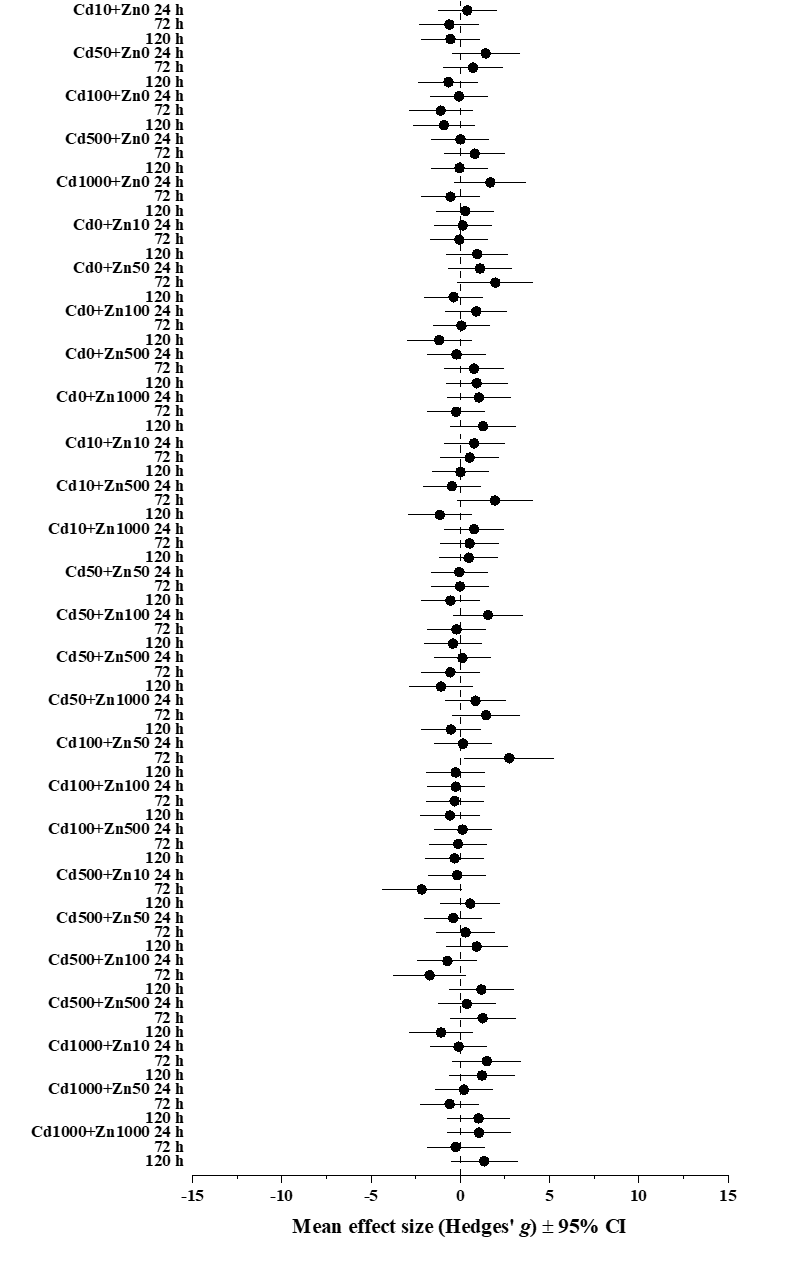


**Supplementary materials B3**. Mean effect sizes (mean Hedges’ *g* ± 95% CI) for the accumulation of K in sorghum in different treatments (mg kg^-1^) after different exposure times (hours). Negative *g* values indicate higher concentration in individuals grown in contaminated vs. ones from uncontaminated media. The mean effect size was considered statistically significant if the 95% bootstrap confidence interval (CI) did not include zero.


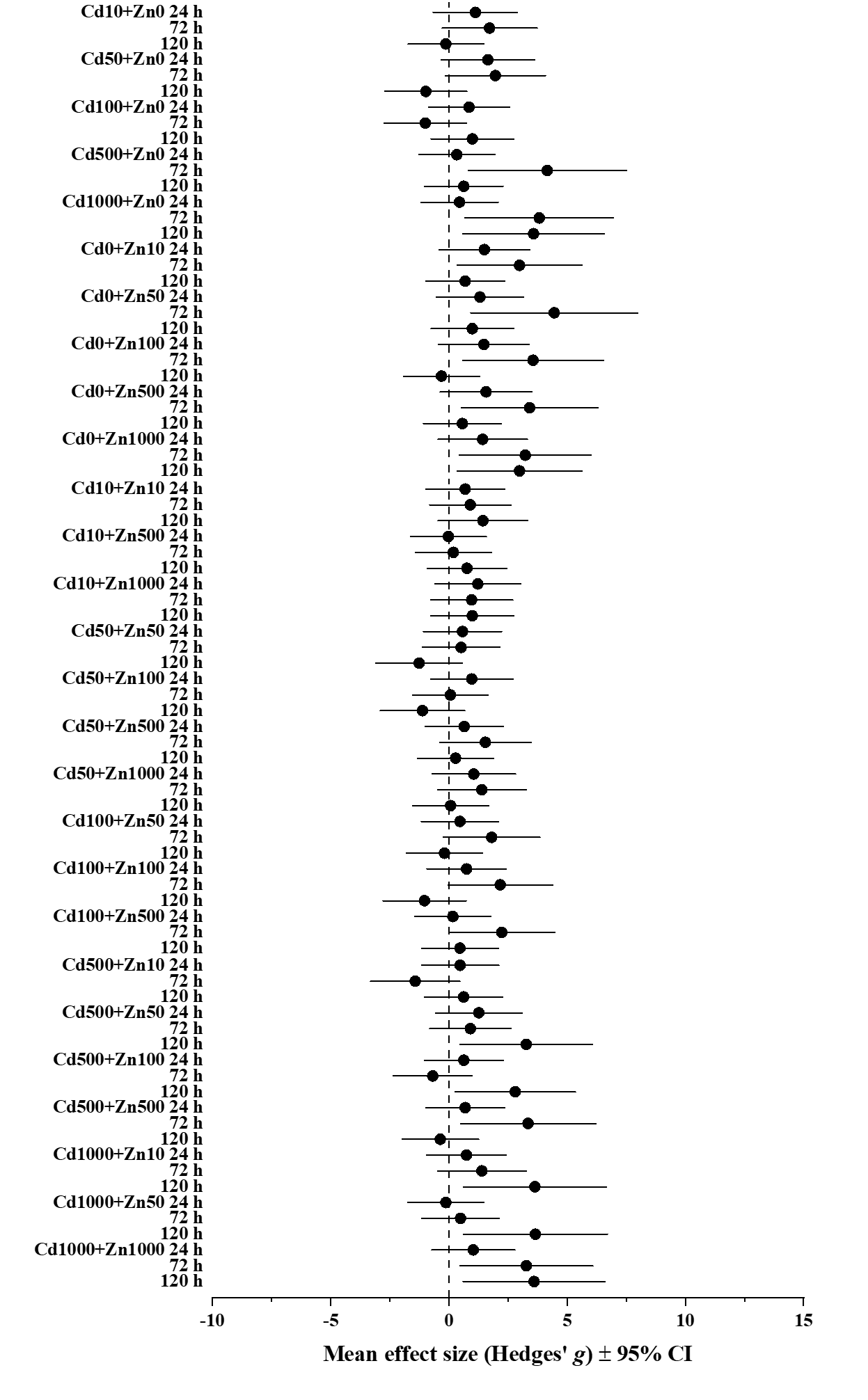


**Supplementary materials B4**. Mean effect sizes (mean Hedges’ *g* ± 95% CI) for the accumulation of K in Sudan grass in different treatments (mg kg^-1^) after different exposure times (hours). Negative *g* values indicate higher concentration in individuals grown in contaminated vs. ones from uncontaminated media. The mean effect size was considered statistically significant if the 95% bootstrap confidence interval (CI) did not include zero.


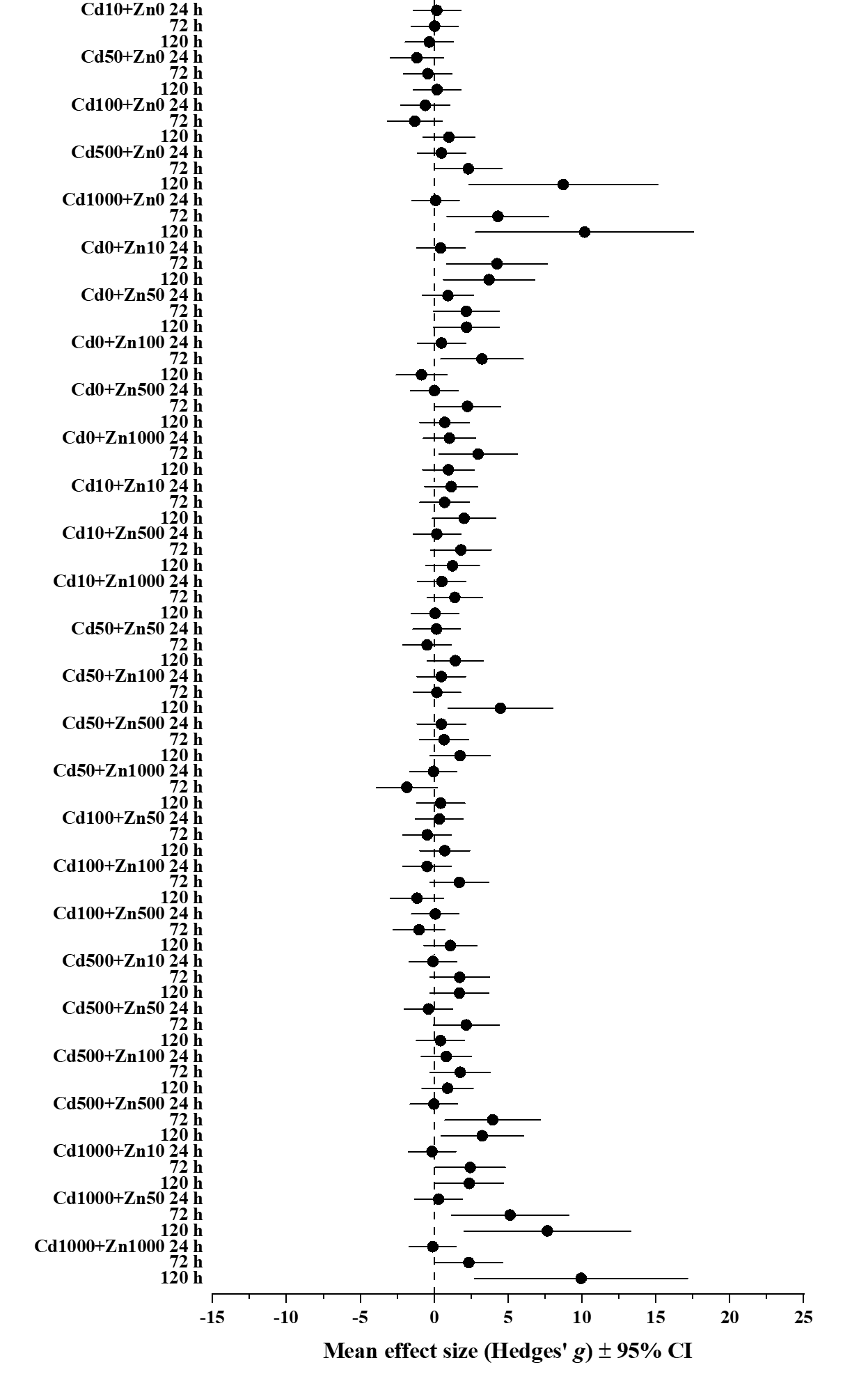


**Supplementary materials B5**. Mean effect sizes (mean Hedges’ *g* ± 95% CI) for the accumulation of Mg in sorghum in different treatments (mg kg^-1^) after different exposure times (hours). Negative *g* values indicate higher concentration in individuals grown in contaminated vs. ones from uncontaminated media. The mean effect size was considered statistically significant if the 95% bootstrap confidence interval (CI) did not include zero.


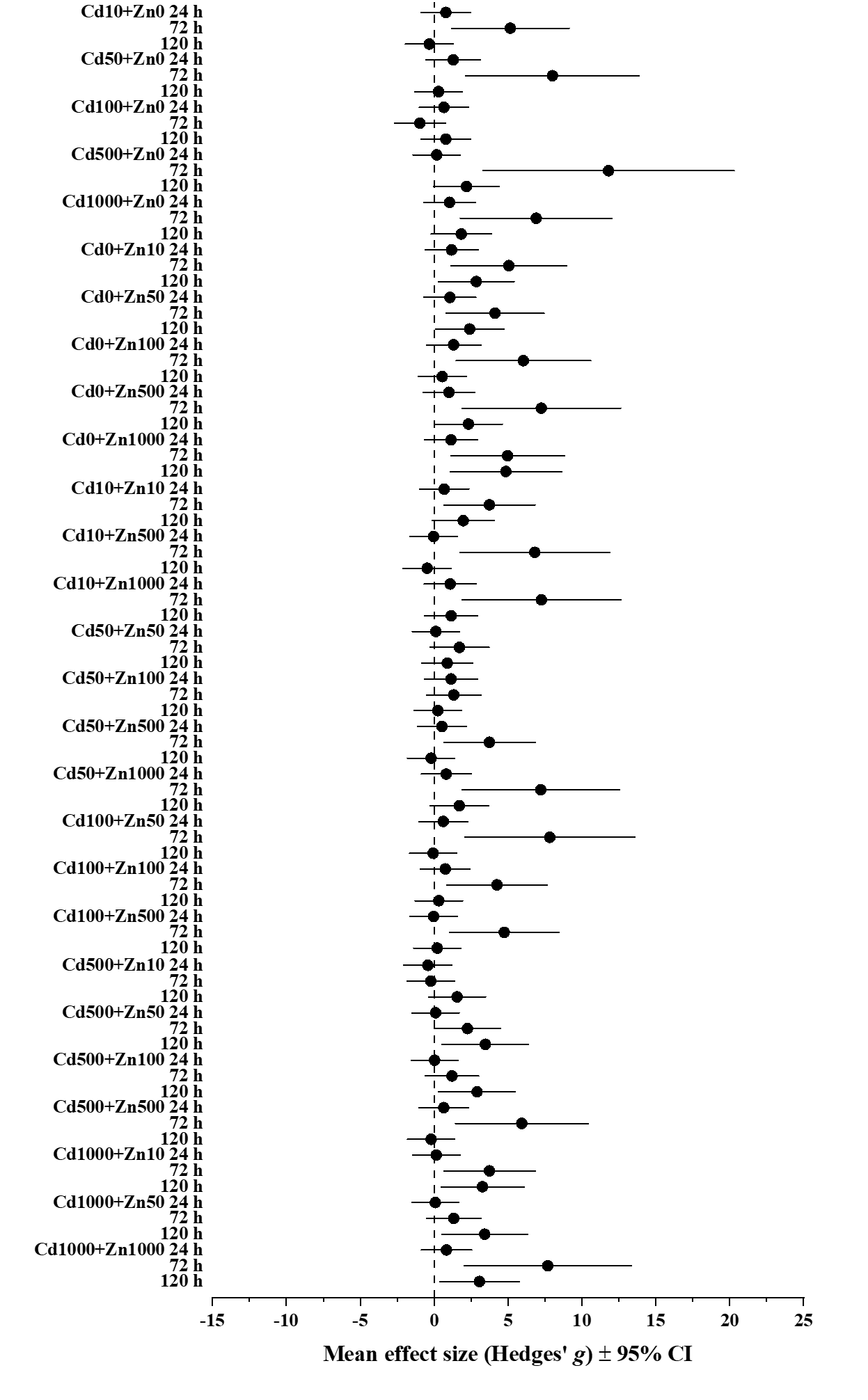


**Supplementary materials B6**. Mean effect sizes (mean Hedges’ *g* ± 95% CI) for the accumulation of Mg in Sudan grass in different treatments (mg kg^-1^) after different exposure times (hours). Negative *g* values indicate higher concentration in individuals grown in contaminated vs. ones from uncontaminated media. The mean effect size was considered statistically significant if the 95% bootstrap confidence interval (CI) did not include zero.


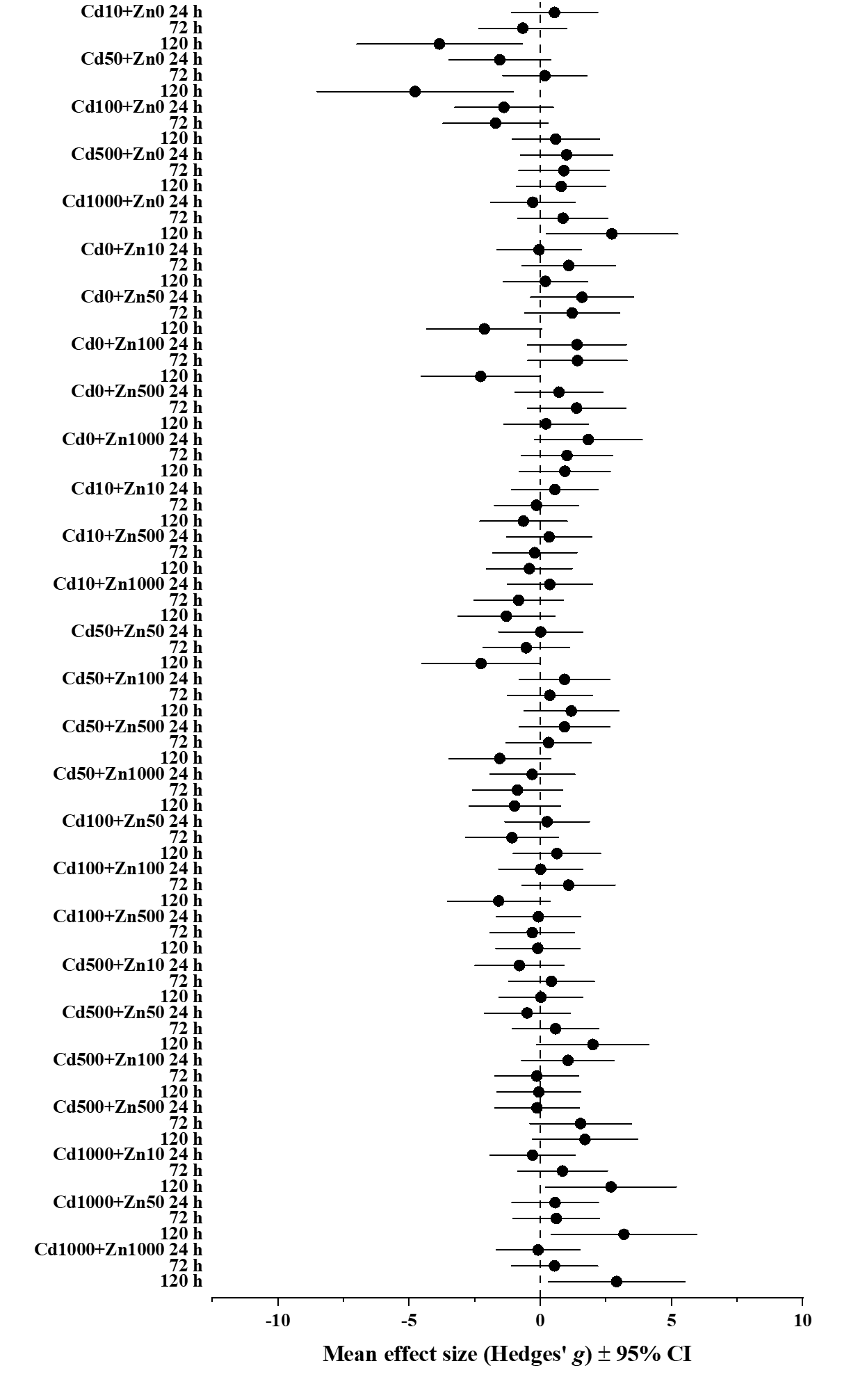


**Supplementary materials B7**. Mean effect sizes (mean Hedges’ *g* ± 95% CI) for the accumulation of Cd in sorghum plantlets in different treatments (mg kg^-1^) after different exposure times (hours). Negative *g* values indicate higher concentration in individuals grown in contaminated vs. ones from uncontaminated media. The mean effect size was considered statistically significant if the 95% bootstrap confidence interval (CI) did not include zero.


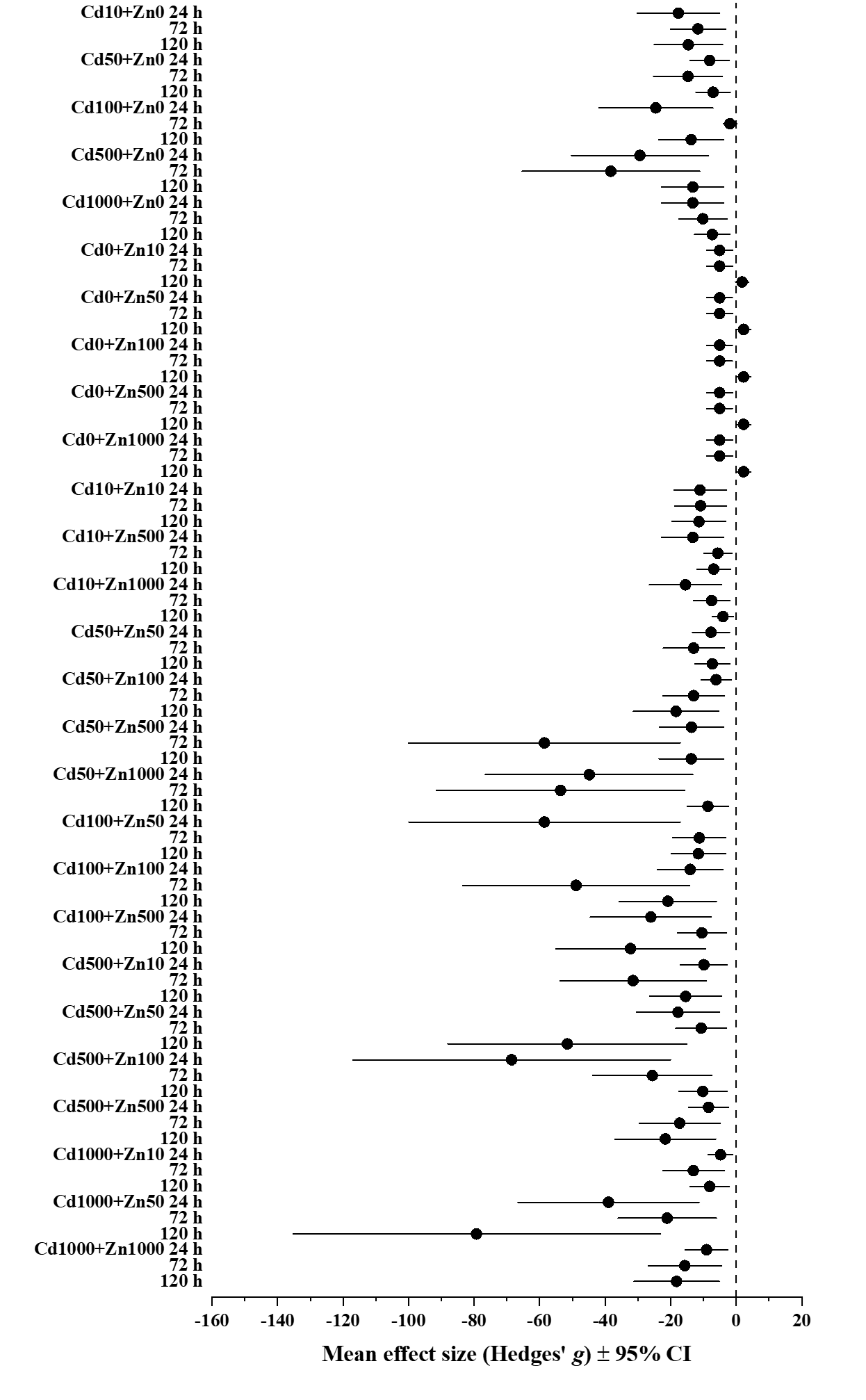


**Supplementary materials B8**. Mean effect sizes (mean Hedges’ *g* ± 95% CI) for the accumulation of Cd in Sudan grass plantlets in different treatments (mg kg^-1^) after different exposure times (hours). Negative *g* values indicate higher concentration in individuals grown in contaminated vs. ones from uncontaminated media. The mean effect size was considered statistically significant if the 95% bootstrap confidence interval (CI) did not include zero.


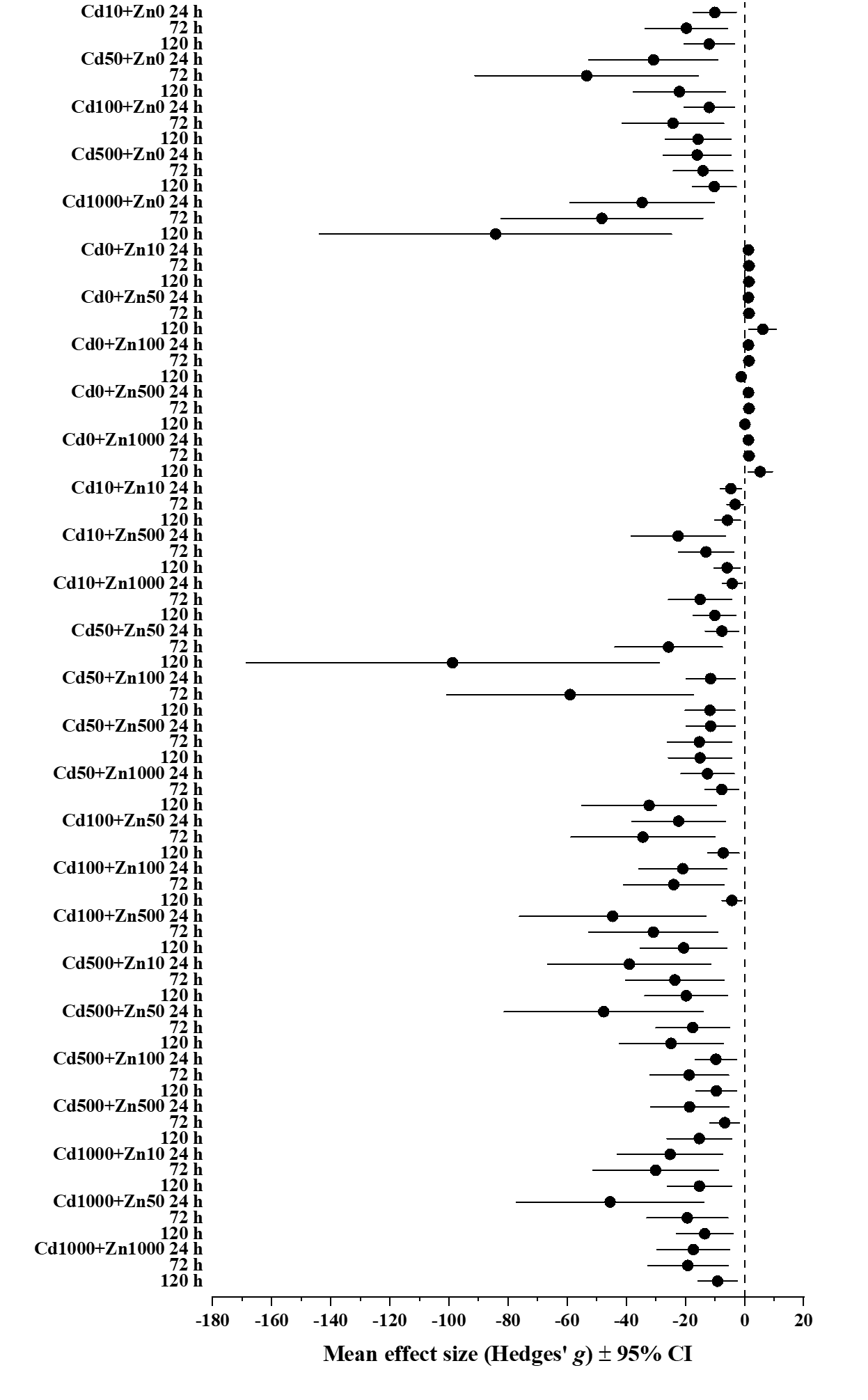


**Supplementary materials B9**. Mean effect sizes (mean Hedges’ *g* ± 95% CI) for the accumulation of Cu in sorghum plantlets in different treatments (mg kg^-1^) after different exposure times (hours). Negative *g* values indicate higher concentration in individuals grown in contaminated vs. ones from uncontaminated media. The mean effect size was considered statistically significant if the 95% bootstrap confidence interval (CI) did not include zero.


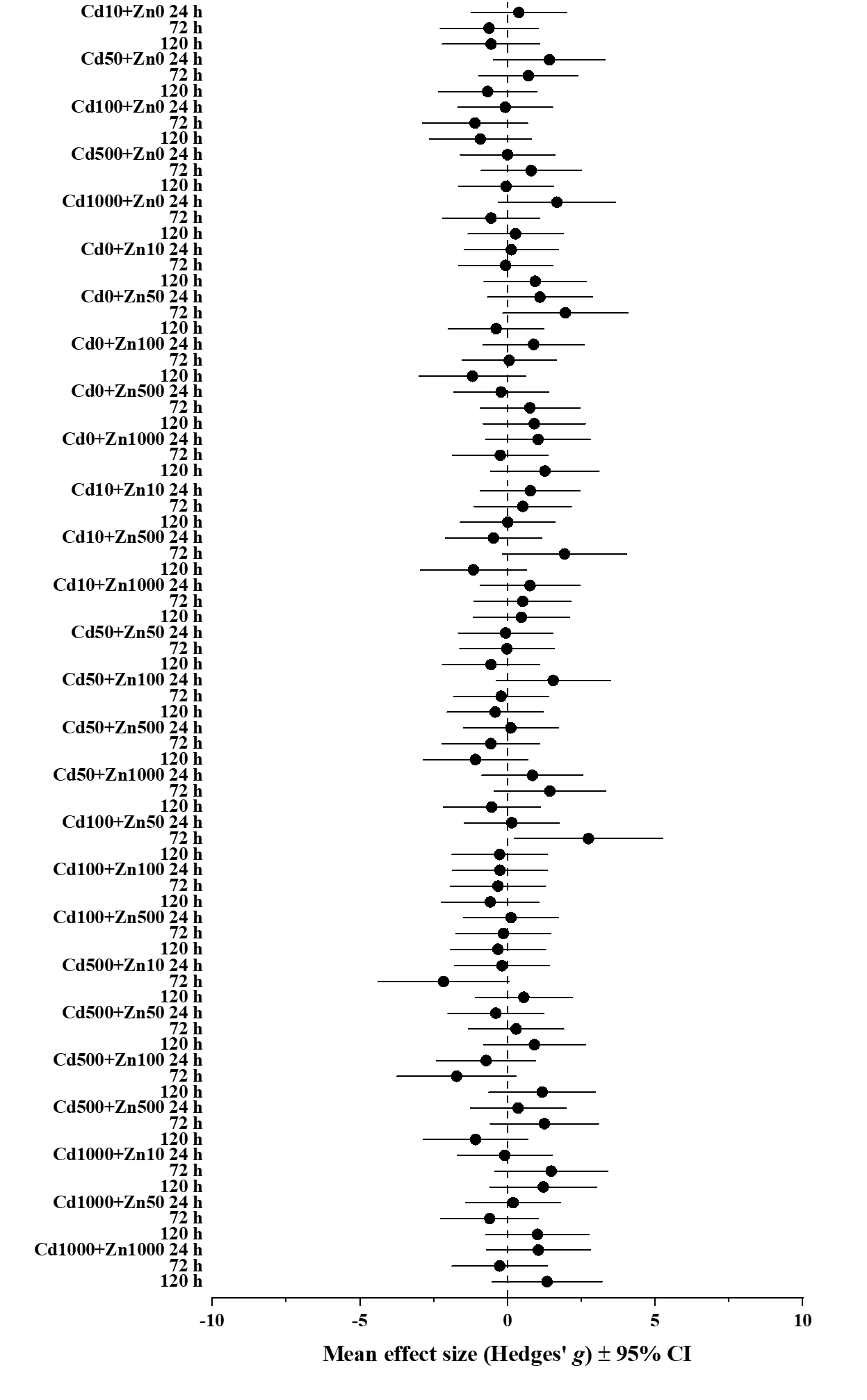


**Supplementary materials B10**. Mean effect sizes (mean Hedges’ *g* ± 95% CI) for the accumulation of Cu in Sudan grass plantlets in different treatments (mg kg^-1^) after different exposure times (hours). Negative *g* values indicate higher concentration in individuals grown in contaminated vs. ones from uncontaminated media. The mean effect size was considered statistically significant if the 95% bootstrap confidence interval (CI) did not include zero.


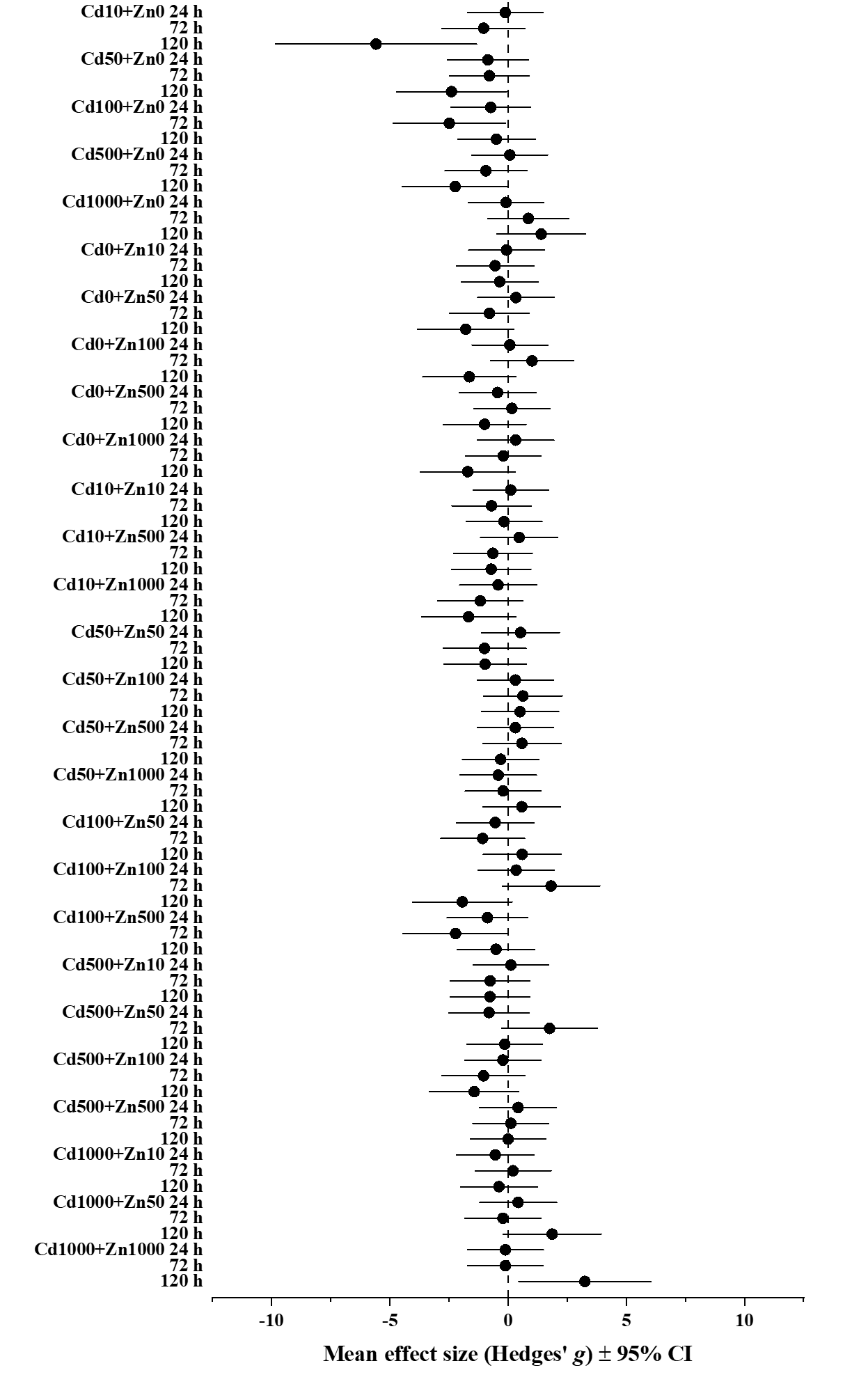


**Supplementary materials B11**. Mean effect sizes (mean Hedges’ *g* ± 95% CI) for the accumulation of Fe in sorghum plantlets in different treatments (mg kg^-1^) after different exposure times (hours). Negative *g* values indicate higher concentration in individuals grown in contaminated vs. ones from uncontaminated media. The mean effect size was considered statistically significant if the 95% bootstrap confidence interval (CI) did not include zero.


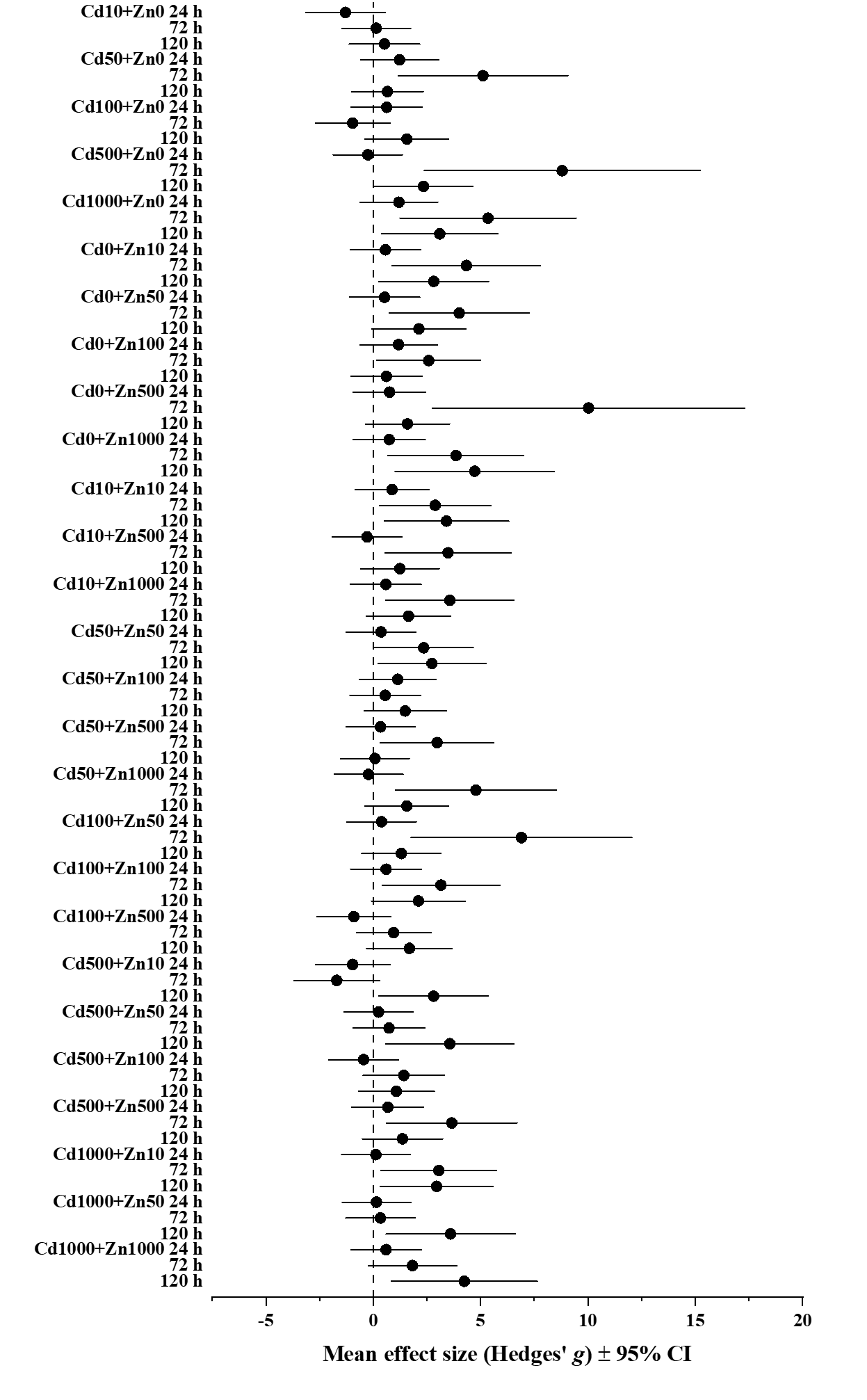


**Supplementary materials B12**. Mean effect sizes (mean Hedges’ *g* ± 95% CI) for the accumulation of Fe in Sudan grass in different treatments (mg kg^-1^) after different exposure times (hours). Negative *g* values indicate higher concentration in individuals grown in contaminated vs. ones from uncontaminated media. The mean effect size was considered statistically significant if the 95% bootstrap confidence interval (CI) did not include zero.


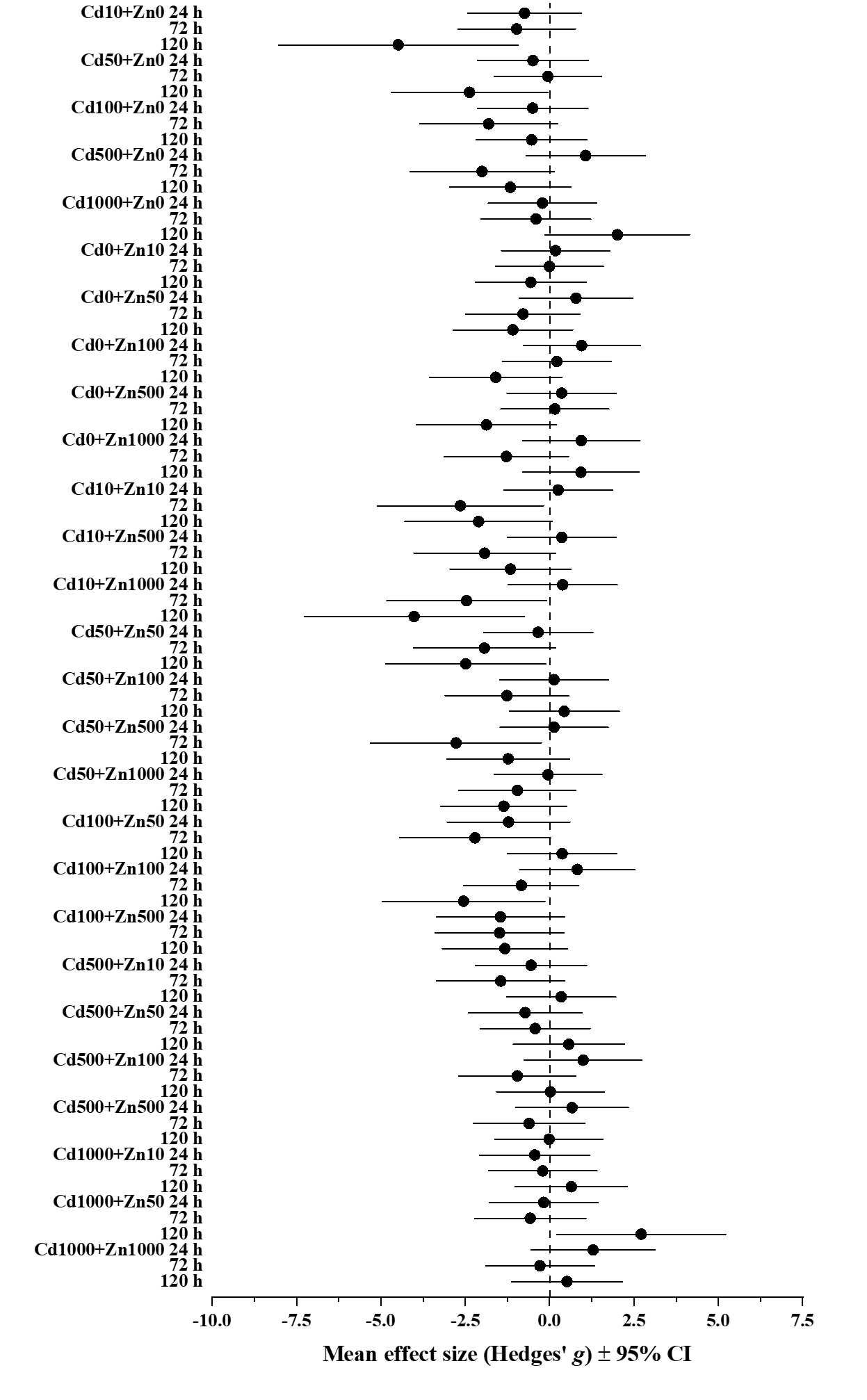


**Supplementary materials B13**. Mean effect sizes (mean Hedges’ *g* ± 95% CI) for the accumulation of Mn in sorghum in different treatments (mg kg^-1^) after different exposure times (hours). Negative *g* values indicate higher concentration in individuals grown in contaminated vs. ones from uncontaminated media. The mean effect size was considered statistically significant if the 95% bootstrap confidence interval (CI) did not include zero.


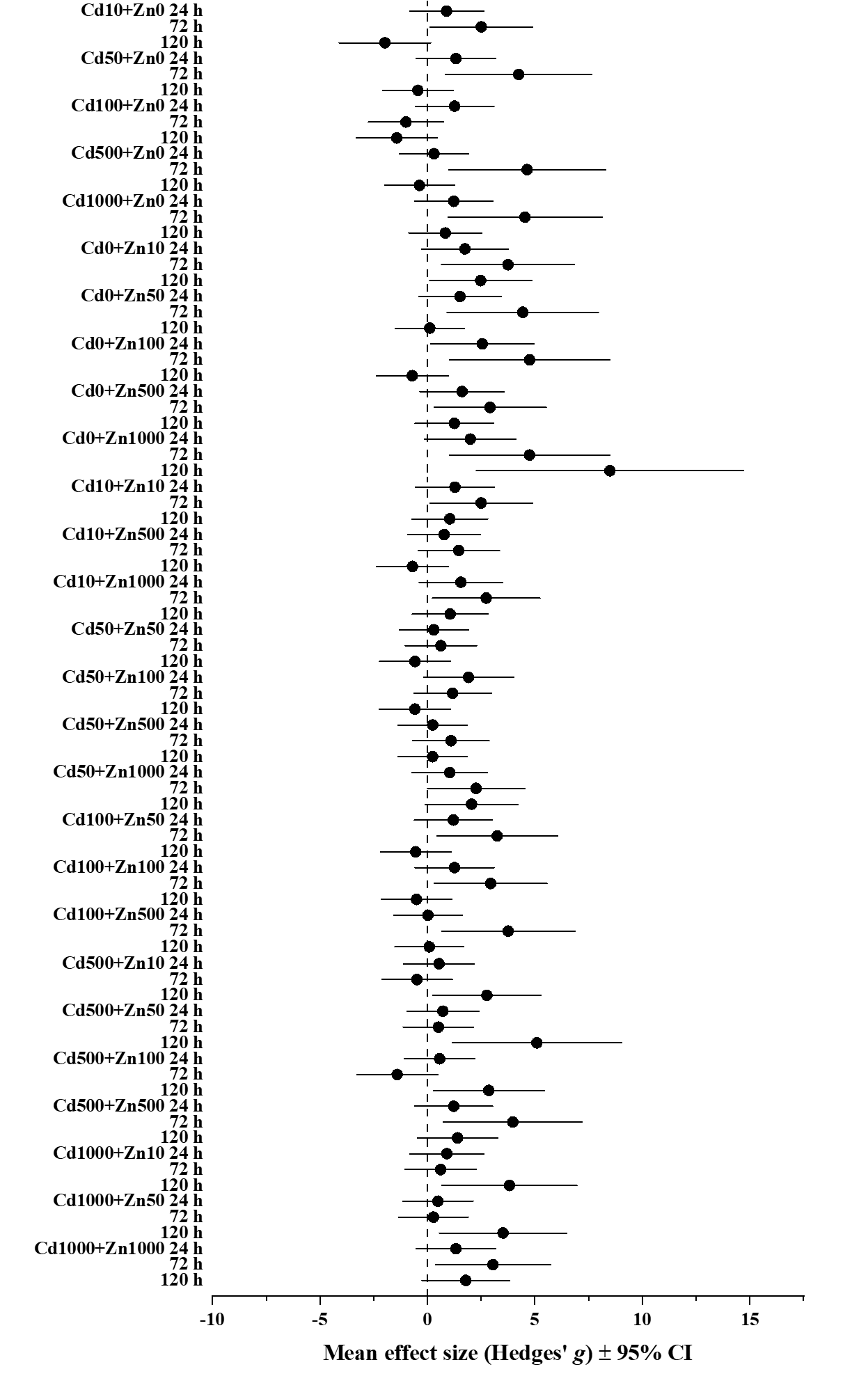


**Supplementary materials B14**. Mean effect sizes (mean Hedges’ *g* ± 95% CI) for the accumulation of Mn in Sudan grass in different treatments (mg kg^-1^) after different exposure times (hours). Negative *g* values indicate higher concentration in individuals grown in contaminated vs. ones from uncontaminated media. The mean effect size was considered statistically significant if the 95% bootstrap confidence interval (CI) did not include zero.


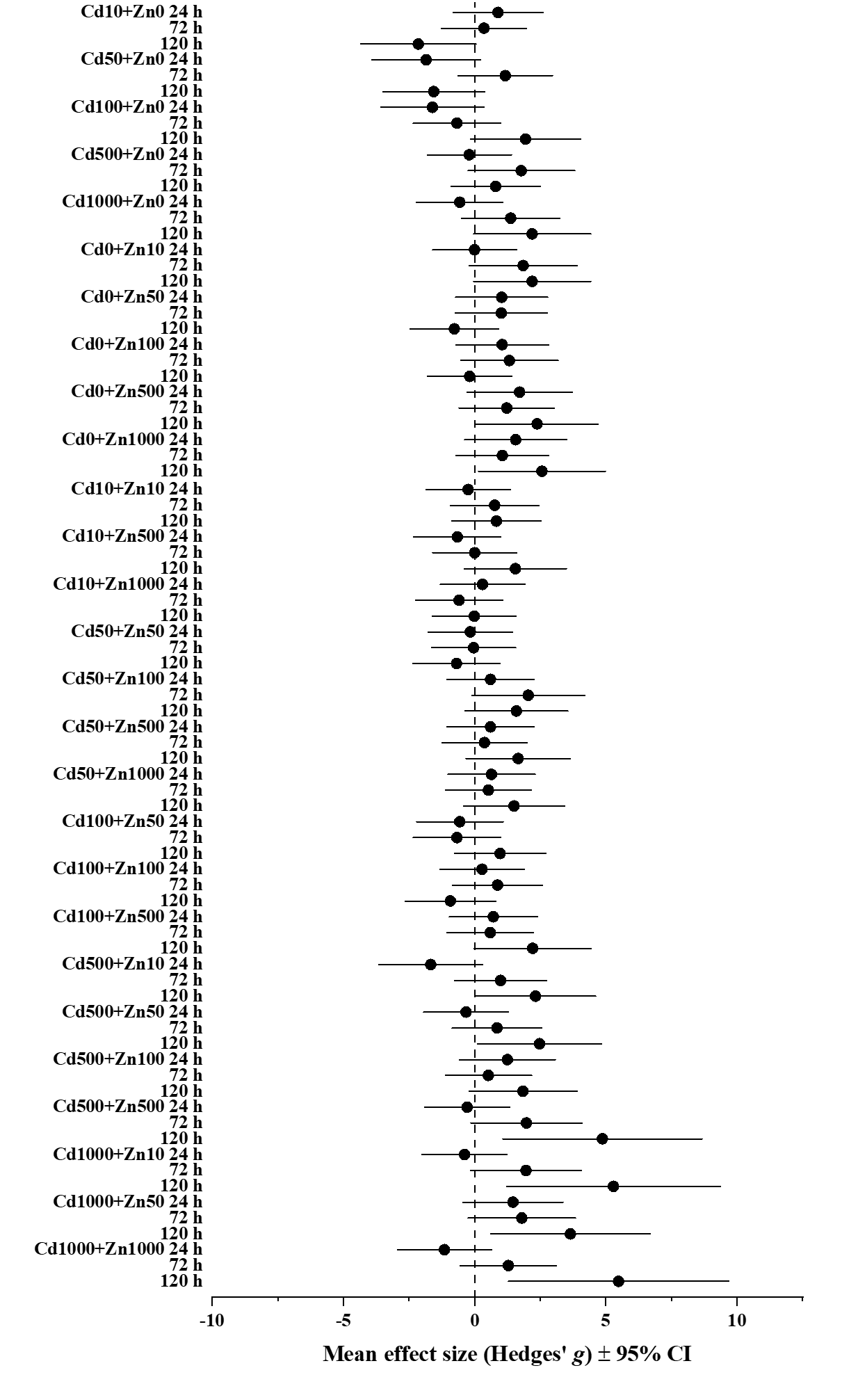


**Supplementary materials B15**. Mean effect sizes (mean Hedges’ *g* ± 95% CI) for the accumulation of Zn in sorghum in different treatments (mg kg^-1^) after different exposure times (hours). Negative *g* values indicate higher concentration in individuals grown in contaminated vs. ones from uncontaminated media. The mean effect size was considered statistically significant if the 95% bootstrap confidence interval (CI) did not include zero.


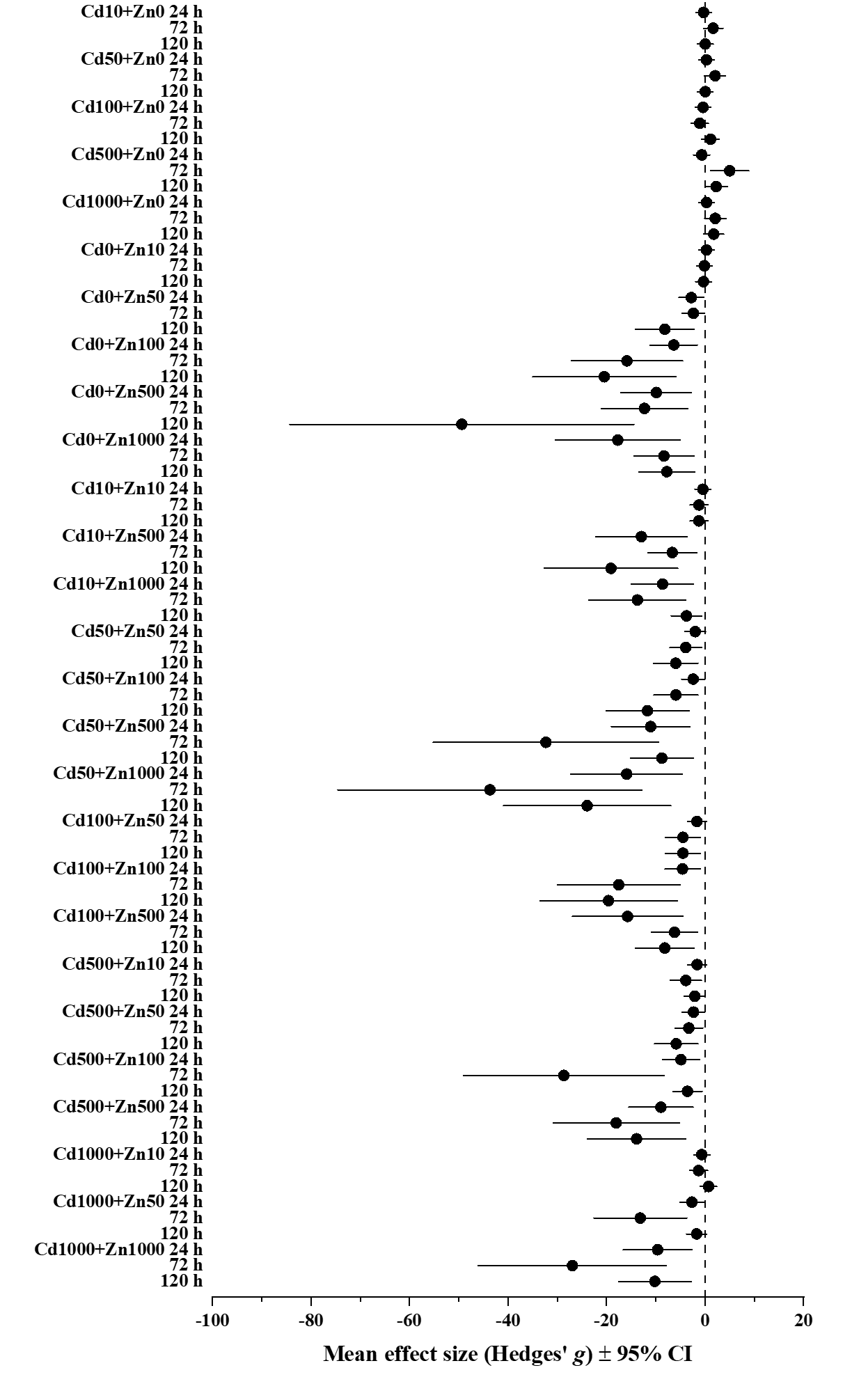


**Supplementary materials B16**. Mean effect sizes (mean Hedges’ *g* ± 95% CI) for the accumulation of Zn in Sudan grass in different treatments (mg kg^-1^) after different exposure times (hours). Negative *g* values indicate higher concentration in individuals grown in contaminated vs. ones from uncontaminated media. The mean effect size was considered statistically significant if the 95% bootstrap confidence interval (CI) did not include zero.


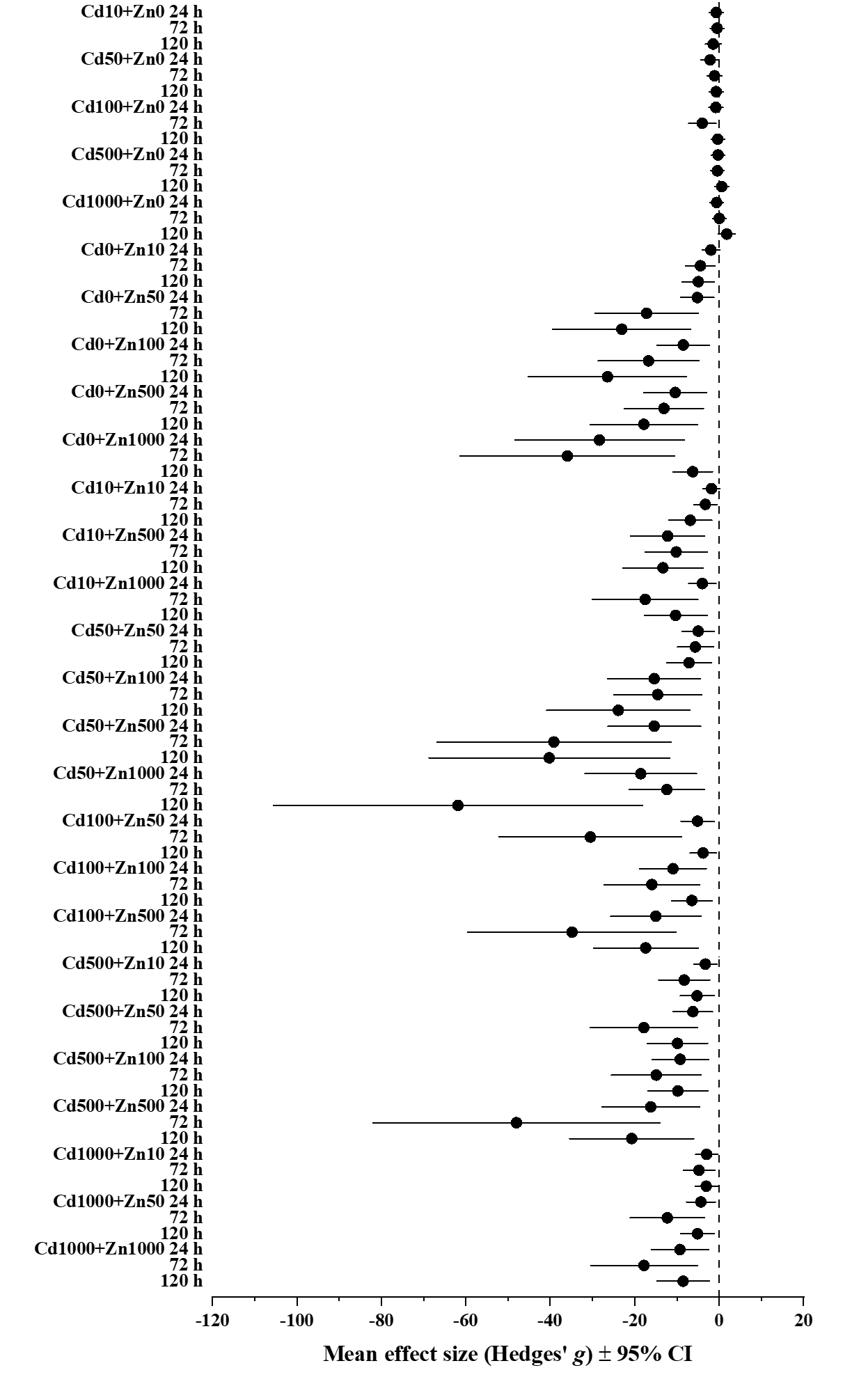


**Supplementary Materials C1**. Interactions between the studied elements in sorghum based on the relationship between the standardized mean difference (Hedges’ *g*) values from single Cd- and single Zn-contaminated vs. uncontaminated comparisons. The linear regression calculations were performed with the pooled mean values of 24-, 72- and 120-h treatments.

| **Interaction** | | **R^2^** | **F** | **dF** | **p** |
| --- | --- | --- | --- | --- | --- |
| *Effect of* | *On* |  |  |  |  |
| Cd | Ca | 0.0076 | 0.0989 | 13 | 0.7582 |
|  | Cu | 0.0935 | 1.3411 | 13 | 0.2677 |
|  | Fe | <0.0001 | 0.0017 | 13 | 0.9680 |
|  | K | 0.0807 | 1.1414 | 13 | 0.3048 |
|  | Mg | 0.0059 | 0.0772 | 13 | 0.7855 |
|  | Mn | 0.0570 | 0.7864 | 13 | 0.3913 |
|  | Zn | <0.0001 | 0.0001 | 13 | 0.9771 |
| Zn | Ca | <0.0001 | 0.0020 | 13 | 0.9648 |
|  | Cd | 0.0999 | 1.4428 | 13 | 0.2511 |
|  | Cu | <0.0001 | 0.0019 | 13 | 0.9660 |
|  | Fe | 0.0044 | 0.0569 | 13 | 0.8153 |
|  | K | 0.1207 | 1.7836 | 13 | 0.2046 |
|  | Mg | 0.0038 | 0.0494 | 13 | 0.8276 |
|  | Mn | 0.0689 | 0.9625 | 13 | 0.3445 |

**Supplementary Materials C2**. Interactions between the studied elements in Sudan grass based on the relationship between the standardized mean difference (Hedges’ *g*) values from single Cd- and single Zn-contaminated vs. uncontaminated comparisons. The linear regression calculations were performed with the pooled mean values of 24-, 72- and 120-h treatments.

| **Interaction** | | **R^2^** | **F** | **dF** | **p** |
| --- | --- | --- | --- | --- | --- |
| *Effect of* | *On* |  |  |  |  |
| Cd | Ca | 0.0489 | 0.6689 | 13 | 0.4282 |
|  | Cu | 0.2675 | 4.7471 | 13 | 0.0483 |
|  | Fe | 0.2930 | 5.3879 | 13 | 0.0372 |
|  | K | 0.0046 | 0.0597 | 13 | 0.8108 |
|  | Mg | 0.0750 | 1.0543 | 13 | 0.3232 |
|  | Mn | 0.1171 | 1.7248 | 13 | 0.2118 |
|  | Zn | 0.1180 | 1.7385 | 13 | 0.2101 |
| Zn | Ca | 0.1084 | 1.5803 | 13 | 0.2308 |
|  | Cd | 0.0647 | 0.9000 | 13 | 0.3601 |
|  | Cu | 0.0094 | 0.1236 | 13 | 0.7308 |
|  | Fe | 0.2357 | 3.9863 | 13 | 0.0673 |
|  | K | 0.0010 | 0.0138 | 13 | 0.9083 |
|  | Mg | 0.0171 | 0.2265 | 13 | 0.6420 |
|  | Mn | 0.0632 | 0.8772 | 13 | 0.3661 |
